# Microbial turnover times in the deep seabed studied by amino acid racemization modelling

**DOI:** 10.1038/s41598-017-05972-z

**Published:** 2017-07-18

**Authors:** Stefan Braun, Snehit S. Mhatre, Marion Jaussi, Hans Røy, Kasper U. Kjeldsen, Christof Pearce, Marit-Solveig Seidenkrantz, Bo Barker Jørgensen, Bente Aa. Lomstein

**Affiliations:** 1 Center for Geomicrobiology, Department of Bioscience, Aarhus University, Ny Munkegade 114, 8000 Aarhus C, Denmark; 2 Section for Microbiology, Department of Bioscience, Aarhus University, Ny Munkegade 114, 8000 Aarhus C, Denmark; 3 Center for Past Climate Studies, Department of Geoscience, Aarhus University, 8000 Aarhus C, Denmark; 4 Department of Geological Sciences and Bolin Centre for Climate Research, Stockholm University, 10691 Stockholm, Sweden; 5 Arctic Research Center, Department of Bioscience, Aarhus University, 8000 Aarhus C, Denmark

## Abstract

The study of active microbial populations in deep, energy-limited marine sediments has extended our knowledge of the limits of life on Earth. Typically, microbial activity in the deep biosphere is calculated by transport-reaction modelling of pore water solutes or from experimental measurements involving radiotracers. Here we modelled microbial activity from the degree of D:L-aspartic acid racemization in microbial necromass (remains of dead microbial biomass) in sediments up to ten million years old. This recently developed approach (D:L-amino acid modelling) does not require incubation experiments and is highly sensitive in stable, low-activity environments. We applied for the first time newly established constraints on several important input parameters of the D:L-amino acid model, such as a higher aspartic acid racemization rate constant and a lower cell-specific carbon content of sub-seafloor microorganisms. Our model results show that the pool of necromass amino acids is turned over by microbial activity every few thousand years, while the turnover times of vegetative cells are in the order of years to decades. Notably, microbial turnover times in million-year-old sediment from the Peru Margin are up to 100-fold shorter than previous estimates, highlighting the influence of microbial activities on element cycling over geologic time scales.

## Introduction

Marine sediments harbour a microbial ecosystem that vertically extends into the seafloor for more than two kilometres in certain regions of the World Ocean^1^. It has been estimated that this ecosystem contains a total of 2.9–5.4 × 10^29^ vegetative cells^2, 3^, the biomass of which is about equal to all microbial biomass in the ocean water^2–4^. It appears that an equal amount of (dormant) bacterial endospores resides in the seabed^5, 6^.

The microorganisms in this deep, buried biosphere feed on organic compounds deposited from the surface photosynthetic world. A steep decline in energy availability with depth in the seabed poses a strong constraint on the community size and metabolic rate of subsurface microorganisms. Consequently, microorganisms in the deep seabed metabolize at extremely low rates^5–8^.

Metabolic activity and cell growth in the deep biosphere are, however, poorly understood. It is unclear, for example, whether the majority of deeply buried microorganisms (*i*) adapts to the low energy flux and thrives under balanced conditions over millions of years with an equilibrated number of cell divisions and cell deaths, or (*ii*) is dormant (spending energy only for maintaining the most essential biomolecules and functions), while a minor subset is active.

In this study, we calculated average turnover times of sub-seafloor microbial biomass in marine sediments using an extensive dataset of sedimentary total and stereo-isomeric amino acid concentrations, vegetative cell numbers, and endospore numbers. Specifically, we combined the experimental data with an improved version of the D:L-amino acid model, which is a mathematical model that has recently been developed to assess microbial activity in subsurface, low-activity environments^5^.

Together with data on the total nitrogen (TN) concentrations in the sediments, the data on the amino acid concentrations were also used to evaluate the quality of marine sedimentary organic matter by the established diagenetic indicator %T_AA_N, which is the fraction of TN present as amino acid-nitrogen (THAA-N)^9, 10^.

New data are presented from four sediment cores located in the Greenlandic Labrador Sea, the Greenlandic Godthåbsfjord, the Iceland Basin and the Faeroe Bank (Suppl. Fig. S1 and Suppl. Table S1). Recalculation of previously published data is included from three stations at the Peru Margin^5^ and two stations in Aarhus Bay, Denmark^6^ (Suppl. Fig. S1 and Suppl. Table S1). The depositional ages of the studied sediments range from a few decades to millions of years.

With this study, we improved a recently developed mathematical model and used new, better-constrained numerical values of the model’s input parameters to estimate microbial turnover times in the energy-limited marine deep biosphere.

## Results

### Abundance of vegetative cells and bacterial endospores, and relative abundance of Bacteria

**Figure 1:**
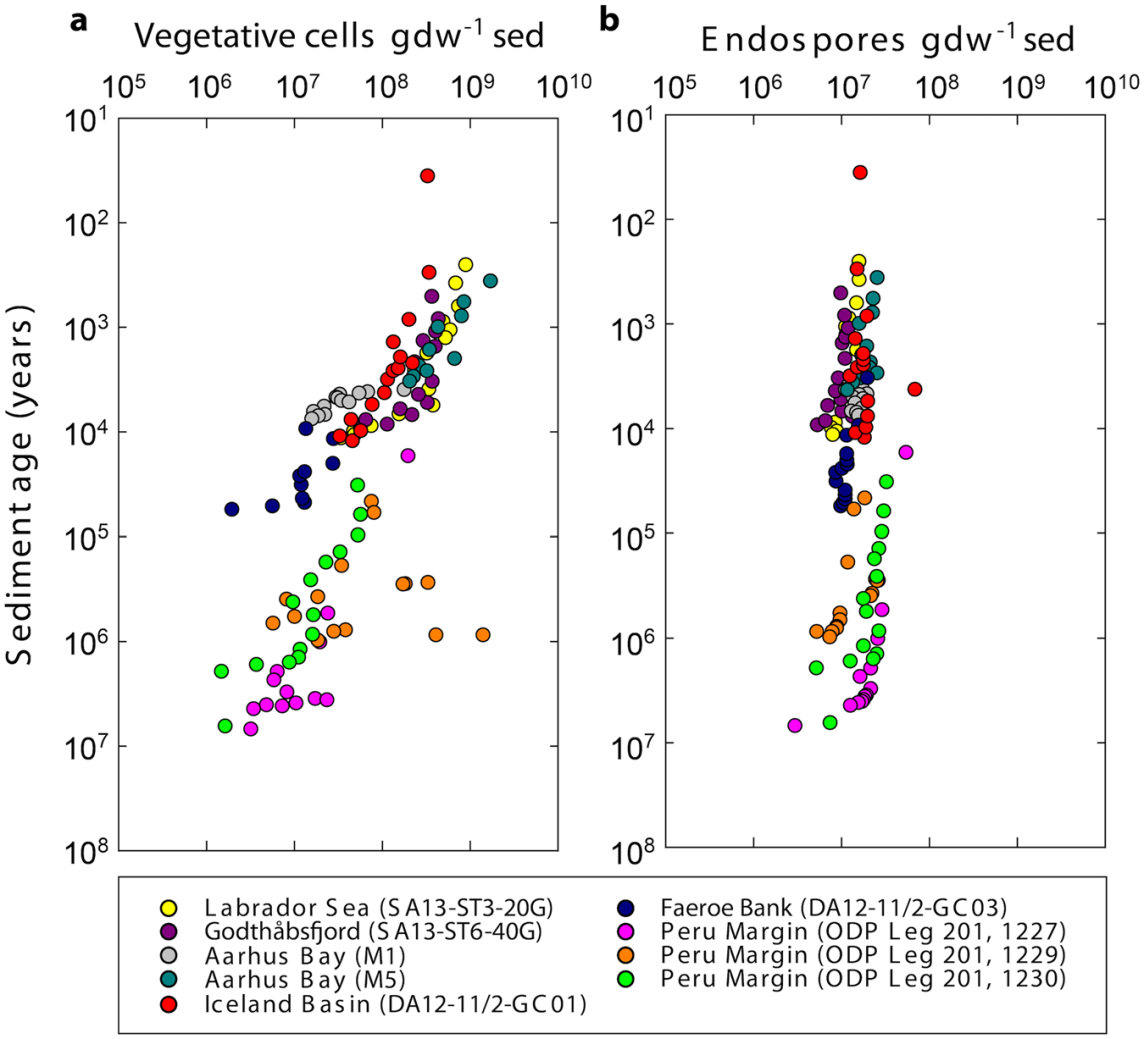
Abundance of vegetative cells (**a**) and bacterial endospores (**b**) versus sediment age. Vegetative cell abundance was determined from epifluorescence microscopy. Endospores were quantified from the bacterial endospore marker dipicolinic acid (DPA). Vegetative cell counts for samples from the Labrador Sea and the Godthåbsfjord were first published in ref. 73. Vegetative cell counts for samples from Aarhus Bay and the Peru Margin are taken from refs 6 and 33, respectively. Endospore data for the Peru Margin and Aarhus Bay are taken from refs 5 and 6, respectively. The unit ‘per gram dry weight sediment’ is abbreviated with ‘gdw^−1^ sed’. Note the logarithmic scales on all axes.

On a double logarithmic scale, the number of vegetative cells decreased roughly linearly with depositional age of the sediment from about 10^9^ vegetative cells per gram dry weight (gdw^−1^) sediment to 10^6^ vegetative cells gdw^−1^ sediment (Fig. 1a). Endospore numbers, estimated from the concentration of dipicolinic acid (DPA), decreased only slightly throughout all sediment cores with a mean abundance of 1.6 × 10^7^ endospores gdw^−1^ sediment (Fig. 1b).

The relative abundance of Bacteria (calculated from qPCR-based quantification of 16S rRNA gene copies in DNA extracted from the sediments as the abundance of Bacteria over the abundance of Bacteria plus Archaea) in the cores from the Labrador Sea (SA13-ST3-20G), the Godthåbsfjord (SA13-ST6-40G) and the Iceland Basin (DA12-11/2-GC01) were between 40% and 80% (Suppl. Fig. S2). At the Faeroe Bank site DA12-11/2-GC03, the relative abundance of Bacteria was near-constant around 40% and reached 60% in the youngest and the two oldest samples. For the sediment cores at the sites M1 and M5 in Aarhus Bay, and sites 1227, 1229 and 1230 from the Peru Margin, no qPCR data were available. For those cores, we assumed equal contributions of Bacteria and Archaea as recently suggested^11^.

### Concentrations of THAA-N and TN

**Figure 2:**
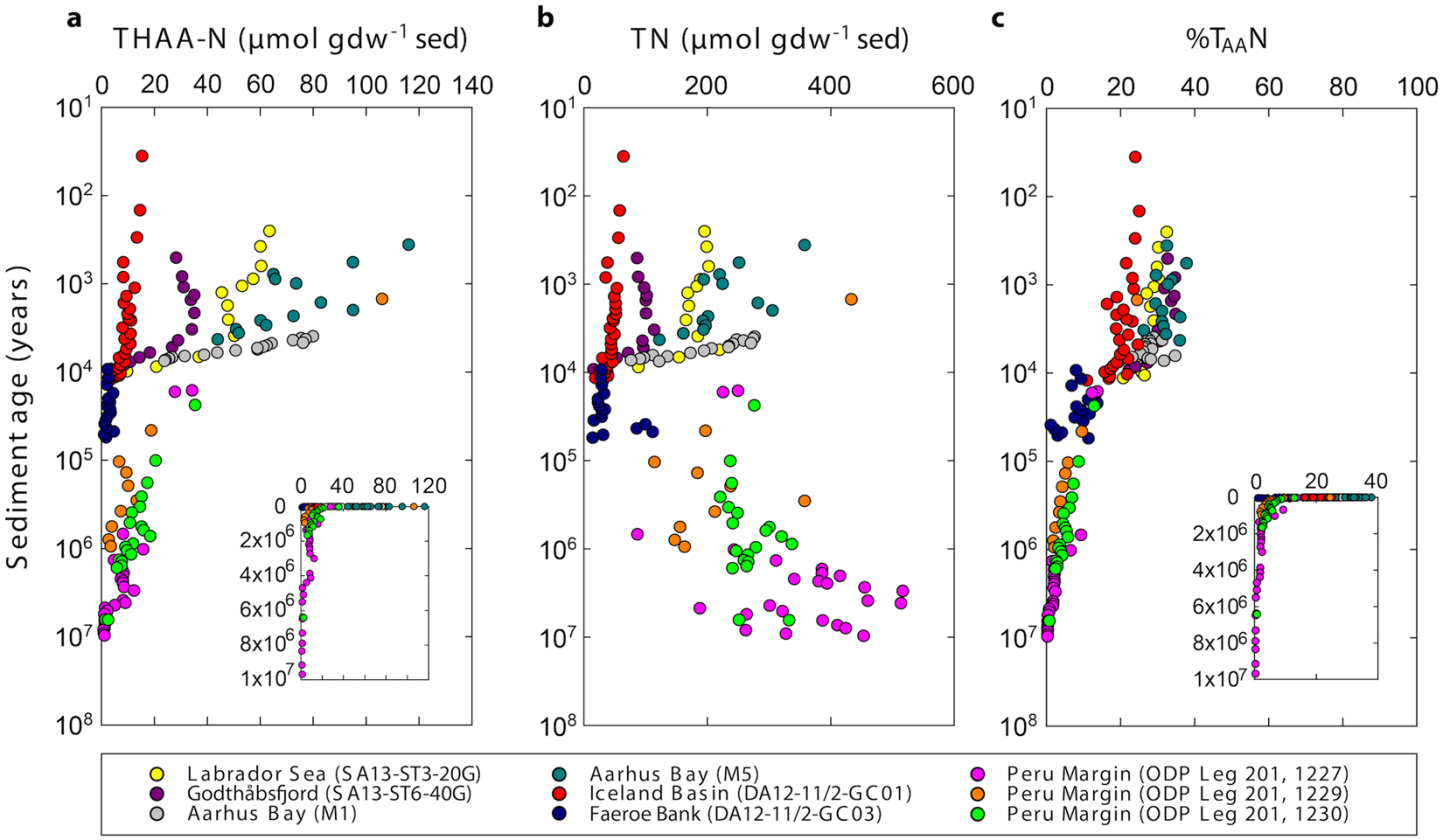
Sedimentary concentrations of total hydrolysable amino acid-nitrogen (THAA-N) and total nitrogen (TN). (**a**) Concentrations of sedimentary THAA-N determined from HPLC analyses of amino acids. (**b**) Concentrations of total nitrogen in the sediment. (**c**) The fraction of total nitrogen present as amino acids (%T_AA_N). The unit ‘per gram dry weight sediment’ is abbreviated with ‘gdw^−1^ sed’. Note that sediment ages are on logarithmic scales. Inserts in (**a**) and (**c**) have linear age scales for better comprehension of the time dimensions.

Concentrations of sedimentary total hydrolysable amino acid-nitrogen (THAA-N) rapidly decreased during the first 10,000 years of burial from up to 100 µmol gdw^−1^ sediment to <10 µmol gdw^−1^ sediment (Fig. 2a). This was followed by a slow decrease to ~1 µmol THAA-N gdw^−1^ sediment in 10 million-year-old sediment.

With the exception of the cores from the Peru Margin, concentrations of total nitrogen (TN) decreased with age from about 400 µmol gdw^−1^ sediment to <50 µmol gdw^−1^ sediment (Fig. 2b). In the organic-rich cores from the Peru Margin, TN was high and varied between 200 and 400 µmol gdw^−1^ sediment.

THAA-N as mole-% of TN (%T_AA_N) is generally recognized as an indicator for the degradation state of organic matter^9, 10^. In young sediment (0–10,000 years), the %T_AA_N rapidly decreased from up to 40% to ~10% (Fig. 2c). In older sediment (up to 10 million years), there was a slow but continuous decrease to values <0.2%.

The concentrations of THAA-N in microbial bio- and necromass (the sum of which is the sedimentary THAA-N) were estimated based on measurements on vegetative cell and endospore abundances and literature conversion factors for the cellular content of THAA-N (see Methods Summary for details on how the calculations were performed). The pool of THAA-N in microbial necromass was 2–3 orders of magnitude larger than that in microbial biomass (Suppl. Fig. S3). Its concentration was only marginally lower than the total sedimentary THAA-N, demonstrating that >95% of total sedimentary THAA-N consists of microbial necromass.

**Figure 3:**
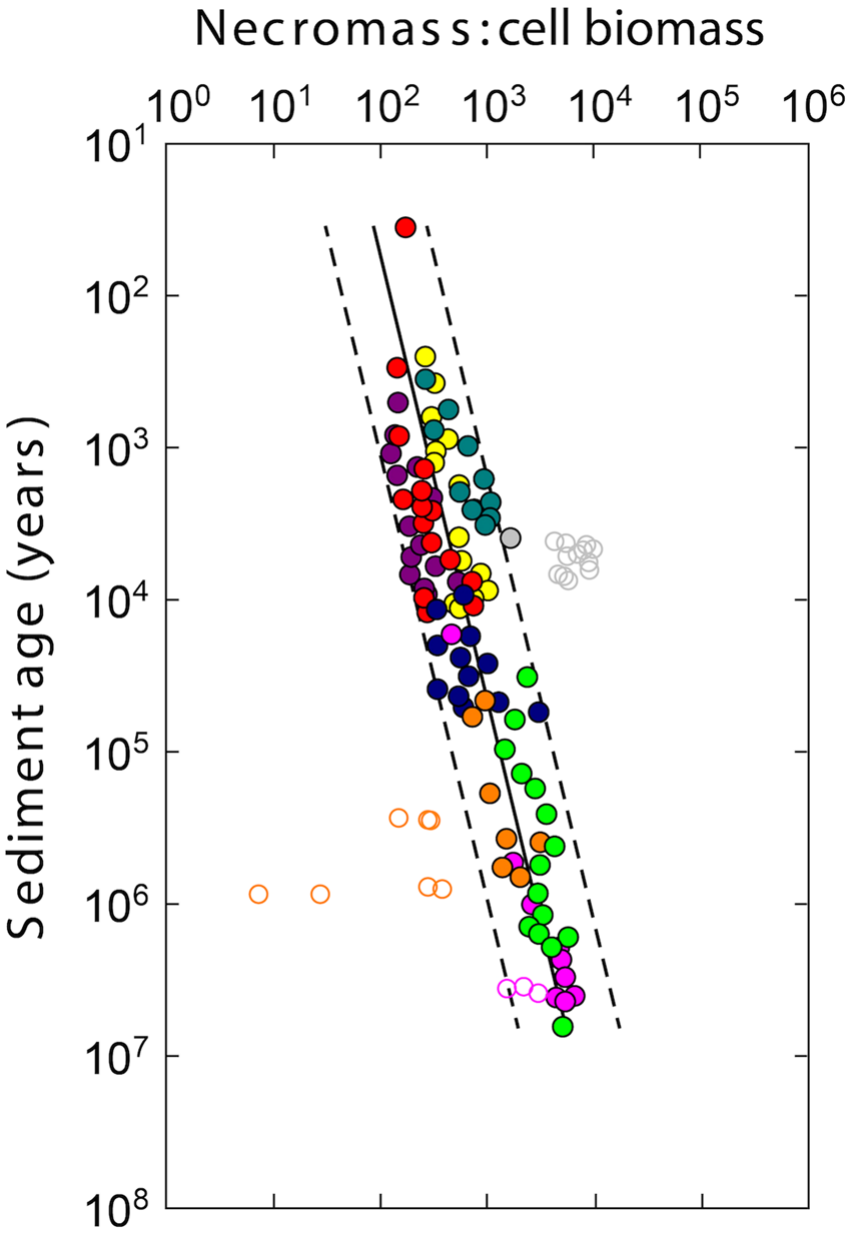
Ratio between THAA-N in microbial necromass and biomass (vegetative cells only). The fraction of THAA-N derived from microbial necromass increases with age relative to that derived from vegetative cells. Note that the concentrations of THAA-N in vegetative cells and necromass were not measured directly, but calculated based on measurements on vegetative cell and endospore abundances and literature conversion factors for the cellular content of THAA-N (see Methods Summary for details on how the calculations were performed). Regression lines show necromass:vegetative cell biomass ratio versus sediment age (solid line; log_10_(necromass:vegetative cell biomass) = 1.405 × log_10_(sediment age) + 0.343, *N* = 98, *R* ^2^ = 0.76, *P* < 0.0001, least squares analysis) and 95% prediction interval (dashed lines). Open circles denote data points that have been removed from regression analysis (e.g. outliers, sulphate-methane transition zones). Note that sediment age is shown on a logarithmic scale. Colour legend is the same as for Figs 1 and 2.

A more detailed assessment of the relative abundance of THAA-N in necromass and vegetative cells is shown in Fig. 3. On a double logarithmic scale, the ratio between THAA-N in necromass and vegetative cells increased linearly with age from ~100 to ~7,000. Note that endospores were not included in this calculation.

On a double logarithmic scale, the abundance of vegetative cells was roughly linearly correlated with the concentrations of THAA-N (*R* ^2^ = 0.54) (Suppl. Fig. S4).

### Occurrence of L-Asp and D:L-Asp ratios

**Figure 4:**
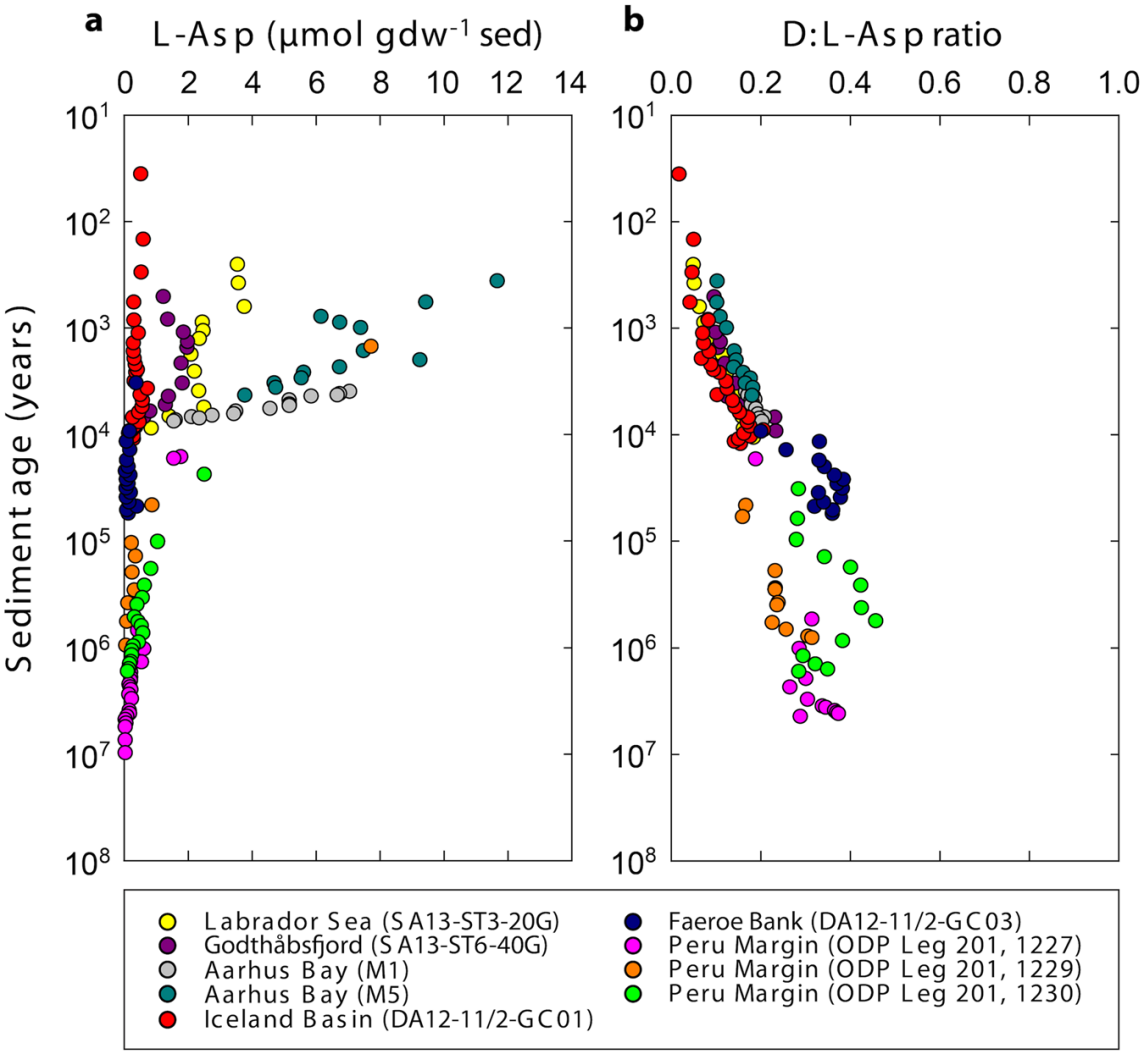
Occurrence of L-Asp and D:L-Asp ratios. (**a**) Total concentrations of L-Asp. (**b**) D:L-Asp ratios increase with age of the sediment. The unit ‘per gram dry weight sediment’ is abbreviated with ‘gdw^−1^ sed’. Note that sediment ages are on logarithmic scales.

**Figure 5:**
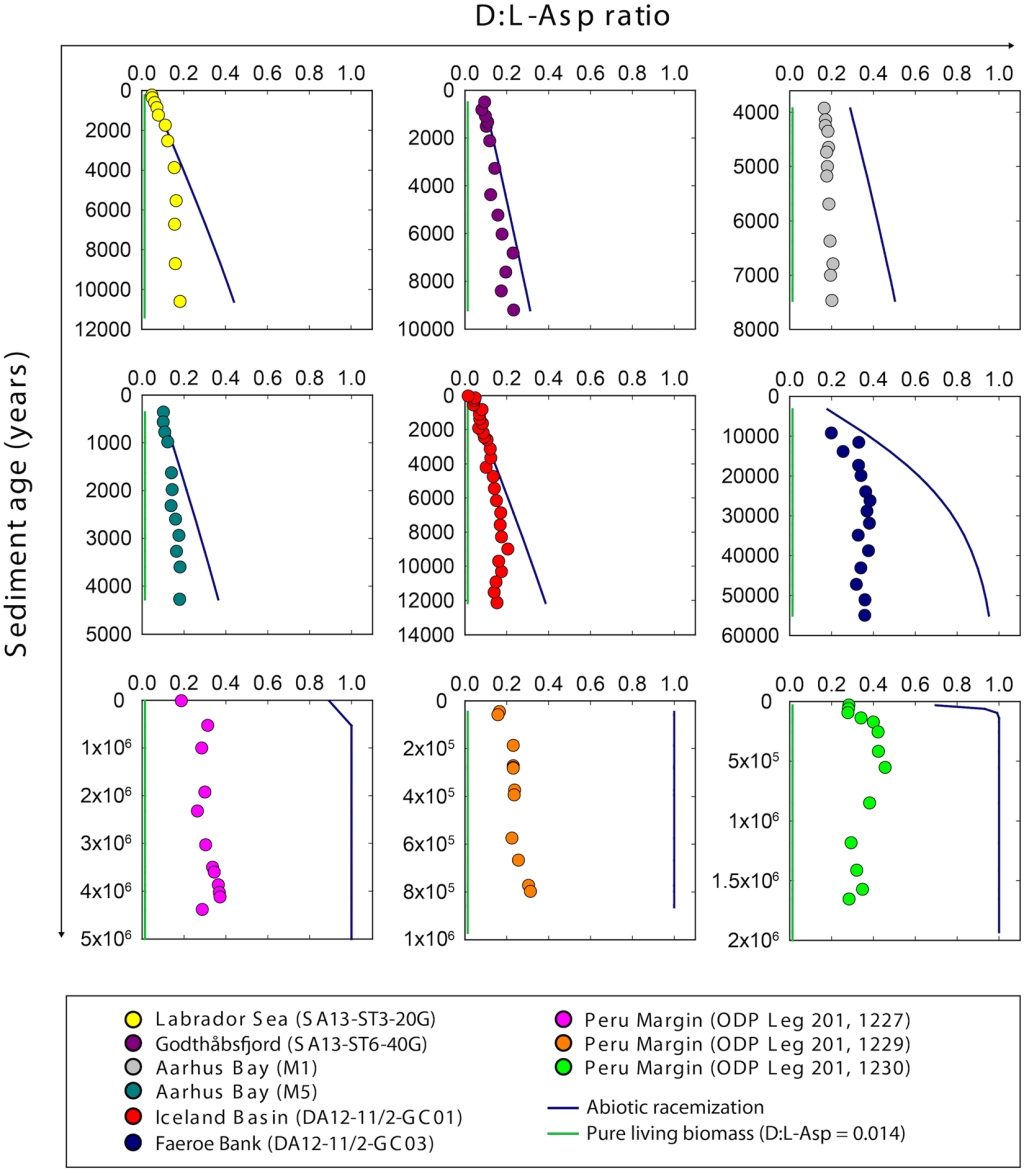
Balance between abiotic racemization and biological turnover of Asp. Ratios of D:L-Asp in the sediment (colored circles) are higher than that of pure vegetative cell biomass (green lines, D:L-Asp ratio = 0.014)^15^, but lower than those predicted from pure chemical racemization (blue lines)^12, 13^. In very old sediments (>10^5^ years), D:L-Asp ratios are between 0.3 and 0.4, whereas pure racemization would have led to a ratio of 1.0.

Concentrations of L-aspartic acid (L-Asp) decreased rapidly from up to 12 µmol gdw^−1^ sediment to ~0.2 µmol gdw^−1^ sediment during the first 10,000 years of burial (Fig. 4a). This was followed by a slow but further decrease to values < 0.02 µmol gdw^−1^ sediment in million-year-old sediment. Throughout all cores, there was a general increase in the abundance of D-Asp relative to L-Asp with increasing sediment age (Fig. 4b). From the youngest to the oldest samples, the D:L-Asp ratios increased by an order of magnitude from <0.05 to 0.4. The ratios were, however, generally lower than those that would have resulted from pure racemization kinetics (Fig. 5), suggesting that microorganisms continuously degrade the partly racemized old necromass and synthesize new L-amino acids. The balance between abiotic chemical racemization and biological turnover of Asp was then calculated using the D:L-amino acid model^5^, which provides estimates on the turnover times of microbial bio- and necromass and on amino acid-carbon (THAA-C) oxidation rates.

### Microbial turnover times and THAA-C oxidation rates

**Figure 6:**
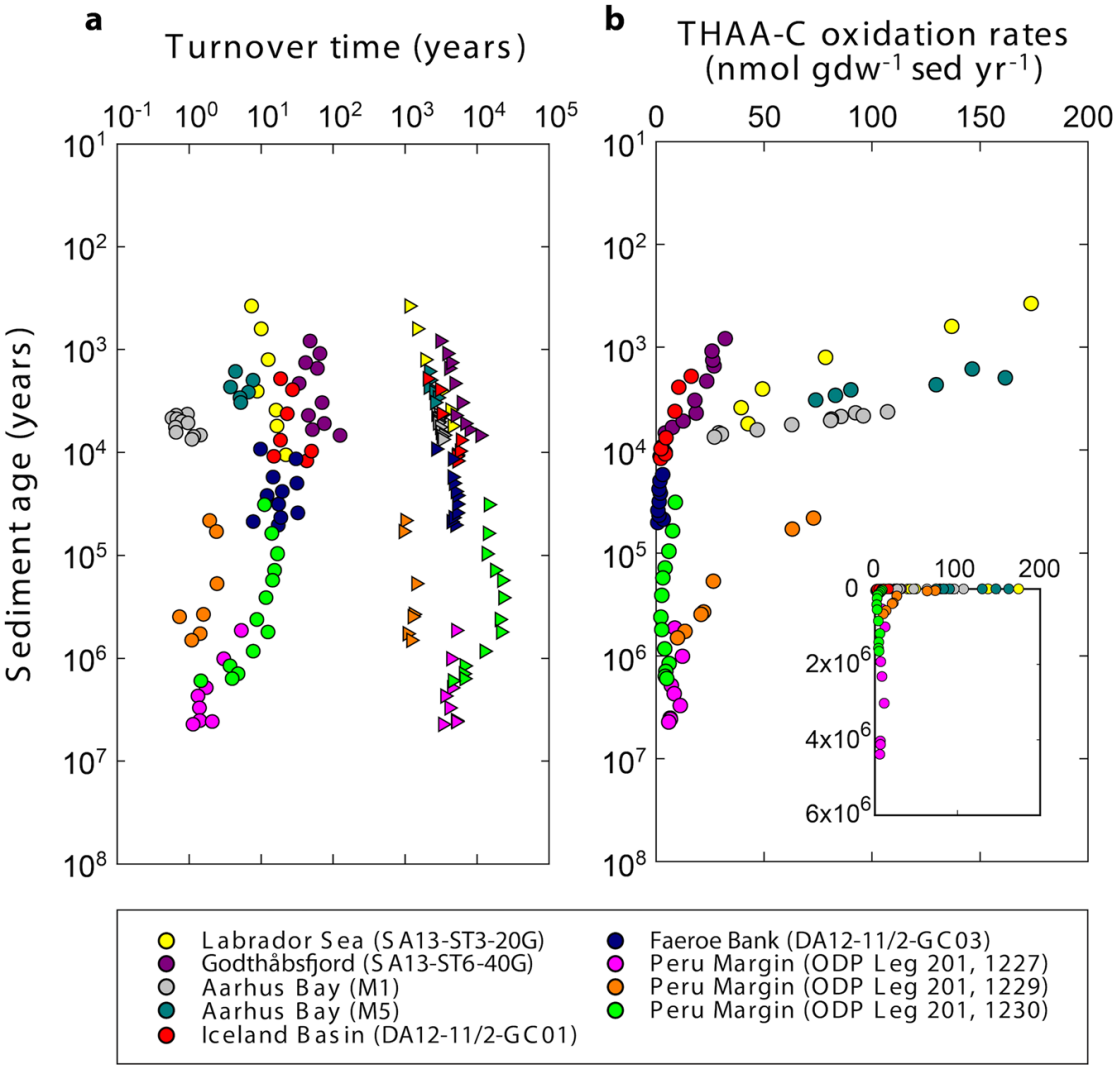
Model-estimated carbon oxidation rates and turnover times of microbial bio- and necromass. (**a**) D:L-amino acid model-estimated turnover times of vegetative cells (circles) and necromass amino acids (triangles) in the seabed. (**b**) Model-estimated oxidation rates of amino acid-carbon (THAA-C) in the samples. The unit ‘per gram dry weight sediment per year’ is abbreviated with ‘gdw^−1^ sed yr^−1^’. Note that sediment ages are on logarithmic scales. Insert in (**b**) has a linear age scale for better comprehension of the time dimensions.

Our new and revised estimates of the turnover times of microbial biomass and necromass in marine sediments showed that necromass amino acids are recycled over hundreds to thousands of years (Fig. 6a). These turnover times were short relative to the age of the sediment. Generally, the necromass turnover times slowly increased with sediment age. With only a few hundreds of years, the necromass turnover times at site 1229 at the Peru Margin were the shortest among the studied cores.

The turnover times of vegetative microbial cells were in the order of years to decades, with maximum values of ~120 years (Fig. 6a). Most of these biomass turnover times were scattered between values of a few years to a few decades. Exceptions were Aarhus Bay M1, which showed very short turnover times of <1 year, and the cores from the Peru Margin, which showed surprisingly short biomass turnover times of <1 to ~17 years.

Using the D:L-amino acid model, we calculated the oxidation rates of the total amino acid-carbon (THAA-C) in the samples according to equation (27) in the Supplementary Information. These rates decreased from up to 200 nmol gdw^−1^ sediment year^−1^ to <10 nmol gdw^−1^ sediment year^−1^ in million-year-old sediment (Fig. 6b). The steepest decrease in THAA-C oxidation rates with sediment age was in samples <10,000 years old.

In this study, we used new data for four input parameters of the D:L-amino acid model, and the model itself was improved by including the racemization back reaction of D- to L-Asp. The effects of these modifications are described below.

(i) We here used a racemization rate constant of Asp, k_i(Asp)_, for protein-bound amino acids^12, 13^, which is about an order of magnitude higher than that for free amino acids used in the initial studies^5, 6^. The k_i(Asp)_ has an inverse linear relationship to both the necromass turnover time, T_NM_, and the biomass turnover time, T_b_. In other words, increasing the k_i(Asp)_ by 10-fold reduces the turnover times of bio- and necromass each by 10-fold. The k_i(Asp)_ has a direct linear relationship with the THAA-C oxidation rates, r_THAA-C_.
(ii) We used a cell-specific THAA-C content for sub-seafloor microorganisms of 1.03 fmol cell^−1^ (ref. 14), which is ~4 times lower than that used in the previous studies^5, 6^. The cell-specific THAA-C content has a direct linear relationship with T_b_. The ~4 times lower cell-specific THAA-C content has therefore resulted in ~4 times shorter biomass turnover times. In contrast, the effect of the cell-specific THAA-C content on T_NM_ and r_THAA-C_ was negligible.
(iii) We used a cell-specific D:L-Asp ratio of sub-seafloor microorganisms of 0.014 (ref. 15) instead of 0.086 (refs 5 and 6). This increased the T_b_ and T_NM_ by a factor of 1.2–2.8. In contrast, the r_THAA-C_ was reduced by 20–60%.
(iv) It turned out that the fraction of Bacteria over Bacteria + Archaea has negligible effects on T_b_, T_NM_ and r_THAA-C_.
(v) Including the racemization reaction from the D- to the L-form of Asp into the model reduced the degradation rate of microbial necromass by 20–45%. Therefore, T_NM_ and T_b_ increased by a factor of up to ~2, while r_THAA-C_ decreased by a factor of up to ~2.

## Discussion

### Endospores in marine sediments

The detection of endospores in marine sediments was until recently limited to cultivation-dependent techniques after sample pasteurization, e.g. refs 16 and 17. However, these techniques have been shown to underestimate endospore abundance by at least three orders of magnitude^18^. New culture-independent approaches have been developed to quantify bacterial endospores^18, 19^. Based on the quantification of DPA, we showed that endospore abundance in the studied marine sediments decreased only slightly over millions of years with a mean value of 1.6 × 10^7^ endospores gdw^−1^ sediment.

The half-life of endospores in cold marine sediments has so far been estimated for thermophilic endospores only^20^. The authors reported half-lives of ~300 years based on the slope of decrease in endospore abundance within 4500 years of burial in Aarhus Bay sediment. Since we here report a near-constant number of endospores over millions of years of burial, these endospores must either have sustained with half-lives far longer than the 300 years estimated by ref. 20, or the endospore abundance must be controlled by a dynamic balance between germination and sporulation during burial.

### Pools of THAA-N and quality of buried organic matter

THAA-N in vegetative cells, bacterial endospores, and microbial necromass was quantified to estimate the contribution of microbial necromass to total sedimentary amino acids and total organic matter. Our data showed that amino acids in microbial necromass are the dominating component of total sedimentary amino acids. With the exception of the sulphate methane transition zones (SMTZs) in sediments from the Peru Margin, microbial necromass accounted for >95% of total THAA-N.

The %T_AA_N decreased with depositional age, indicating a gradual shift in the balance between microbial amino acid consumption and production towards consumption (Fig. 2c). This is because total uncharacterized organic matter typically becomes more and more recalcitrant with age^21–23^, while amino acids remain a labile, more degradable component of total organic matter, e.g. refs 24 and 25. It was reported that sinking particles are more depleted in THAA-N than plankton^26^, especially when they are incorporated into the sediment^24, 25, 27^. This shows that the preferential degradation of amino acids over total organic matter already starts in the water column and that total organic matter becomes progressively depleted in amino acids with increasing age. Hence, deeply buried microorganisms need to break down an increasing proportion of lower-quality compounds to gain energy. Consistent with this notion, we found a power law relationship between the concentrations of vegetative cells and THAA-N (Suppl. Fig. S4). This probably indicates that the microbial community size is controlled by the concentrations of amino acids and other high-quality (more degradable) organic compounds rather than by the total concentration of uncharacterized organic matter.

### Amino acid racemization modelling of microbial activity

The D:L-Asp ratio in the sediments gradually increased with age (Fig. 4b) as the predominant L-form in necromass amino acids was slowly converted towards an equilibrium 1:1 mixture of the D- and the L-form by random abiotic racemization^28^. However, the D:L-Asp ratios presented here were lower than those that would have resulted from pure racemization kinetics (Fig. 5). This shows that there is a continuous turnover of necromass amino acids. Microorganisms continuously degrade the partly racemized old necromass and synthesize new L-amino acids. Microorganisms may also rebuild proteins directly from the necromass amino acids^29^. For this, D-amino acids need to be converted into L-forms by racemases^30^. The continuous reworking of the necromass pool pulls the sedimentary D:L-Asp ratio away from the equilibrium ratio that would otherwise result from pure abiotic racemization (Fig. 5). As a consequence, it is not possible to use amino acid enantiomeric ratios for geochronology^25, 31, 32^. The ubiquity of microbial life in marine sediments means that D:L-amino acid ratios are always influenced by microbial decomposition and bacterially-derived organic matter^12^. For example, the D:L-Asp ratio at site 1230 from the Peru Margin is ~0.4 at a sediment age of ~175,000 years. When we calculate the sediment age based on the sedimentary D:L-Asp ratio (0.4), the D:L-Asp ratio in pure biomass (0.014)^15^, the *in-situ* sediment temperature (2.4 °C)^33^, and the corresponding k_i(Asp)_ (2.9 × 10^−5^)^12, 13^ according to the formulation presented in ref. 28, the sediment age would be ~14,000 years, which is more than ten times less than the actual age of the sediment. These results clearly indicate that biological processes are interfering with pure chemical racemization of L- and D-Asp in the sediment.

Our new and revised estimates of the turnover times of microbial biomass and necromass in marine sediments showed that the pool of microbial necromass is dynamic. There is a constant production of necromass from the decay of the vegetative population and at the same time a degradation of necromass delivering energy and nutrients to the active microbial population. The necromass turnover reported here was two orders of magnitude faster than our previous estimates^5, 6^, highlighting the influence of microbial activities on element cycling over geologic time scales.

The turnover times of vegetative microbial cells were much longer than typical doubling times of microorganisms in laboratory cultures (hours to days, Fig. 6a) or other nutrient-rich environments^34^. However, the recalculated turnover times for the cores from Aarhus Bay and the Peru Margin were up to two orders of magnitude shorter than the initial estimates^5, 6^. This is mainly due to the use of a racemization rate constant for protein-bound amino acids instead of free amino acids. Protein-bound amino acids racemize about an order of magnitude faster than free amino acids^35–37^. Since the D:L-Asp ratios in the marine sediments were lower than those that would have resulted from pure racemization kinetics, microbial reworking of Asp in necromass must proceed on timescales shorter than those predicted from the racemization rate constant of Asp at the *in-situ* temperatures. The racemization rate constants for several (protein-bound)-amino acids including Asp has recently been determined at elevated temperatures using whole sediment from Aarhus Bay^12, 13^. The rates were then extrapolated to the low *in-situ* temperatures (1–16 °C) of the studied sediment cores. The majority of amino acids in marine sediments are protein-bound^38–40^. For example, in the million-year-old sediments from the Peru Margin, dissolved free amino acids measured in pore water were <1% of THAA^40^. We therefore concluded that it was appropriate to use a racemization rate constant for protein-bound amino acids in marine sediments for our samples. Notably, the racemization rate constant for Asp used in this study was similar to that reported for other environments at similar temperatures, e.g. refs 41–43.

The cell-specific carbon content used in this study was approximately four times lower than that used in the initial estimates, which further reduced the turnover times. The available data on subsurface cellular microbial biomass is limited, but consistently suggest that the deep biosphere harbours low-biomass microorganisms. We used a cell-specific THAA-C content of 1.03 fmol cell^−1^, determined for sub-seafloor microbial cells from the Baltic seabed. By assuming that amino acids contained 55% of total cell carbon^44^, this number translated into a total cell-specific carbon content of 23 fg C cell^−1^. Using the same approach, ref. 15 reported a calculated cell-specific carbon content of microorganisms in marine sediment in the Labrador Sea of 19 fg C cell^−1^. Based on cell volumes determined by fluorescence microscopy for sediment samples from the oligotrophic South Pacific Gyre, ref. 2 estimated that the cells had a mean cell-specific carbon content of 14 fg C cell^−1^ (the conversion from volumes to carbon contents was established from relationships between cell size and protein content of pelagic bacteria)^45^.

Based on these data, we conclude that it is appropriate to use a lower cell-specific carbon content (23 fg C cell^−1^) than that used in the initial estimates (~88 fg C cell^−1^)^5, 6^.

Finally, the inclusion of D- to L-Asp racemization in the model further enhanced the calculated turnover by a factor of up to 2.

The biomass turnover times in the studied sediment cores vary by up to two orders of magnitude between sites. This is mainly due to the different temperatures at the sites. Higher temperatures translate into higher racemization rate constants, reducing the turnover times in our model calculations. The sediment core from the Godthåbsfjord showed the longest biomass turnover times, consistent with its low bottom-water temperature of ~2 °C (the lowest mean temperature of the studied cores). At the other end of the spectrum were the cores from the Peru Margin, which had *in-situ* temperatures of up to 16 °C. The high amount of TN (and organic matter in general) in these cores seems to provide sufficient energy for increased microbial activity. While bottom-water temperatures of ~7 °C were recorded at both sites in the Aarhus Bay, they showed biomass turnover times that differed by one order of magnitude. At Aarhus Bay Station M1, cell abundance was about an order of magnitude lower than at Station M5, while the other model input parameters had similar numerical values. Apparently, the fewer cells at Station M1 seem to be as efficient as the total cells at Station M5 in keeping the D:L-Asp ratio lower than that expected from pure chemical racemization. For that, because they are lower in number, they have to have a faster turnover than the cells at Station M5.

Our biomass turnover times from the Peru Margin sites 1227 and 1230 of 1–17 years are close to those by ref. 46, who reported turnover times for the same sites of 0.25–7 years based on sulphate reduction measurements and global estimates of carbon fluxes. In contrast, ref. 47 calculated biomass turnover times in SMTZs from the Peru Margin of ~100–2,000 years based on ecosystem-level carbon budgets. These highly different results were, however, derived from very different assumptions and different parts of the sediment column. For example, the assumed growth efficiencies differed by 10–50 fold, and in ref. 47 only samples from SMTZs were used. Furthermore, areal metabolic rates were calculated per m^2^ by ref. 46, but per cm^2^ by ref. 47. Since the metabolic activity of microorganisms is typically highest at the sediment surface, using areal activity rates per m^2^ will result in average cumulative rates that are higher than those obtained by using areal activity rates per cm^2^.

The D:L-Asp profiles and the relatively short vegetative cell turnover times shown here have several implications and raise questions: first, they illustrate that sub-seafloor microbial communities play an important role in the “recycling” of necromass amino acids. Since major elements such as sulphur and nitrogen are involved in many of the microbially mediated redox reactions^48^, deeply buried microorganisms are substantially influencing the global element cycles^3, 48, 49^. Second, could the fast vegetative cell turnover times be driven by the lytic cycle of virus infections? A recent study indicated active phage-host interactions with both Bacteria and Archaea in the deep seabed^50^. Could the relatively large amount of microbial necromass (compared to biomass) in the sediment be a result of virus-mediated lysis of vegetative cells that eventually releases organic compounds back into the microbial loop? We cannot answer these questions here, but they will be a point of future investigations. Third, the short vegetative cell turnover times resulted in high carbon oxidation rates, which raises questions about how microorganisms are able to oxidise the increasing proportion of recalcitrant uncharacterized organic matter in the deep seabed.

Because of the energy loss (e.g. as heat) intrinsic to metabolic energy conservation reactions^51–53^, the energy and nutrients obtained from the degradation of THAA-C cannot be completely used for cell maintenance or the conversion into new cell material. Therefore, microorganisms additionally need to degrade uncharacterized total organic carbon (TOC) to maintain a steady state community size. Since it was difficult to put a number on the amount of TOC oxidized, we showed minimum carbon oxidation rates presented by the D:L-modelled degradation rates of THAA-C (Fig. 6b). We compared the THAA-C oxidation rates accumulated with depth and age in the sediment to the surface TOC concentrations for each core. Cumulative THAA-C oxidation rates were calculated according to ref. 54. Except for the million-year-old cores from the Peru Margin, cumulative THAA-C oxidation rates for each core showed that <30% of the surface TOC concentration of the corresponding core was oxidized. In cores from the Peru Margin, cumulative THAA-C oxidation was 10-fold higher than the surface TOC concentration. High C-oxidation rates are consistent with fast vegetative cell turnover times. However, C-oxidation rates in the samples from the Peru Margin were apparently overestimated based on the comparison between the cumulative C-oxidation rates and the actual TOC concentrations, suggesting that microbial activity is lower than estimated here. In contrast, the low D:L-Asp ratios in microbial necromass suggest relatively high microbial activity (fast turnover) (Fig. 5). The reason for this discrepancy is unclear. TOC concentrations in the samples from the Peru Margin are, however, not in steady state. Most of the sub-surface samples in the cores have higher TOC concentrations than the core’s surface-most sample. Therefore, surface TOC concentrations millions of years ago may have been much different from today’s. Comparing cumulative C-oxidation to the surface TOC concentrations today may thus be misleading.

We are aware that our calculations of the turnover times of microbial bio- and necromass are mean values, and one could argue that among the total microbial population only a minority of vegetative cells are highly active, whereas the majority is dormant. According to our model results, the deeply buried microorganisms in the Peru Margin have surprisingly fast turnover times of 1–17 years, comparable to surface sediments. If only a minor fraction (e.g. 1–10%) of the total vegetative cells were responsible for the fast turnover of the necromass amino acids (as indicated by the relatively low D:L-Asp ratios), the turnover times for these cells would be days to months. This is similar to the turnover times of sulphate reducing bacteria in pure culture, e.g. refs 55–58. For the Peru Margin and possibly other subsurface environments, such fast turnover times are highly unlikely given the energy-limited conditions of the deep seabed. We therefore suggest that necromass is produced by the total microbial community rather than by a subset. The turnover of the microbial community must include cell division and cell death. If there were no production of new necromass, the total necromass amino acid pool would quickly become degraded and the D:L-Asp ratios of microbial necromass would approach 1.0 in the old sediments from the Peru Margin. Since this is not the case, the microbial population has to be in a state of dynamic equilibrium, where cell growth and division balance mortality.

In conclusion, our data show the combined result of long-term microbial degradation processes and a slowly decreasing community size. With depositional age of the sediment, the balance between microbial amino acid consumption and production gradually shifts towards consumption because amino acids (proteins and peptides) remain more degradable than the total uncharacterized organic matter, which typically becomes more and more recalcitrant with age. The community size is limited by the concentrations of sedimentary amino acids and possibly other high-quality organic compounds. We demonstrate that microorganisms in the deep seabed turn over their biomass on annual to decadal timescales, feeding on the remains of dead cells and uncharacterized total organic matter. Bio- and necromass turnover times are up to two orders of magnitude shorter than previous estimates, suggesting microbial metabolic and physiologic (pre-)adaptations to the low availability of energy^59^. These could, for example, include an extremely low cell-specific energy demand^60^, a small cell body size^14^, and an enhanced, GASP (growth advantage in stationary phase)-like ability to degrade amino acids and total uncharacterized organic matter, e.g. refs 61 and 62.

## Methods Summary

### Sediment material

A map showing the sediment core locations and an overview of their coordinates, water depths, and core lengths can be found in Suppl. Fig. S1 and Suppl. Table S1, respectively. Recalculation of published data was performed on samples obtained from Peru Margin ODP Leg 201^5^ and Aarhus Bay^6^.

New sediment cores were obtained from the following four sites: the Iceland Basin (DA12-11/2-GC01)^63^, the Faeroe Bank (DA12-11/2-GC03)^63^, the continental shelf in the Labrador Sea (SA13-ST3-20G)^64^, and the fjord Kapisigdlit Kanderdluat (SA13-ST6-40G), which forms a part of the Godthåbsfjord complex in SW-Greenland^64^.

At Iceland Basin site DA12-11/2-GC01, the measurement of the bottom water temperature was not successful, but it had previously been reported that the water temperature near this site in the Iceland Basin at ~2000 m water depth is ~3 °C^65^. We here use 3 °C as the temperature for the racemization rate constant of Asp. The bottom water temperature at site DA12-11/2-GC03 was 7.6 °C. The bottom water temperatures at the sites SA13-ST3-20G and SA13-ST6-40G were 4.4 °C and 1.8 °C, respectively.

The age-depth relationships for all four cores are based on AMS radiocarbon measurements of mollusks (SA13-ST3-20G), marine macroalgae (SA13-ST6-40G), planktonic foraminifera (DA12-11/2-GC01 and DA12-11/2-GC03) and pteropods (DA12-11/2-GC01). All ^14^C dates were calibrated with the Marine13 radiocarbon calibration curve^66^ and a local marine reservoir correction ΔR of 0 years, using the Oxcal v4.2 software^67, 68^. The radiocarbon chronologies indicate that the top of all the cores are of modern age and span the last approximately 12,500 calender years Before Present (cal. years B.P.) in core SA13-ST3-20G, 9,500 cal. years B.P. in core SA13-ST6-40G, 12,000 cal. years B.P. in core DA12-11/2-GC01, and up to 55,000 cal. years B.P. in core DA12-11/2-GC03.

Sub-samples were taken from the central part of the sediment cores with sterile cut-off syringes through windows cut into the core liners. Sediment for analysis by high-performance liquid chromatography (HPLC, ~5 cm^3^) was immediately frozen at −20 °C. Before further processing, samples were freeze-dried and homogenized by grinding in an agate mortar. Sediment for cell counting (1 cm^3^) was transferred into centrifuge tubes containing 4 ml filter-sterilized NaCl solution (30 g l^−1^) amended with 2% (v:v) paraformaldehyde. Samples were shaken to form a homogenous slurry, and then stored at 4 °C until further analysis.

### Analysis of amino acids, amino sugars, dipicolinic acid, and TN

These compounds were analysed for D:L-amino acid modelling of microbial activity, and for establishing the diagenetic indicator %T_AA_N and the source indicators Gly:Ser and GlcN:GalN. Concentrations of total hydrolysable amino acids and amino sugars were analysed by HPLC according to the method described by ref. 69 and with the modifications described in ref. 6. Concentrations of D- and L-isomers of amino acids were analysed by HPLC following the method described by ref. 70 and with the modifications described in ref. 71. The acid hydrolysis treatment of the samples deaminates asparagine (Asn) and glutamine (Gln) into aspartic acid (Asp) and glutamic acid (Glu), respectively. We do not know how much of the measured Asp and Glu was initially present as Asn and Gln, respectively, and we therefore cannot distinguish between these different forms. Asp and Glu are here equal to Asp + Asn and Glu + Gln, respectively. This has, however, no impact on the D:L-amino acid modelling results because the measurements of the Asp racemization rate constant, k_i(Asp)_, were also performed on samples that were treated by acid hydrolysis^12, 13^. Dipicolinic acid concentrations were analysed by HPLC and calibrated by standard additions to samples according to ref. 19 with the exception that columns used were a Waters CORTECS^TM^ C18 (2.7 µm; 4.6 × 150 mm) column guarded with a Waters CORTECS® C18 (2.7 µm; 5 × 21 mm) column. Further, the concentration of Tb^3+^ added to standards and samples was increased from 5 µM to 40 µM. The concentration of DPA in each sample was calculated by the use of DPA standard addition to samples together with an unamended sample. The conversion from DPA to endospore numbers is based on the endospore specific DPA content of 2.24 × 10^−16^ mole per endospore^18^.

Before analysis of TN (and total organic carbon, TOC, which was measured contemporaneously), any traces of inorganic C were removed by treating the sediment samples overnight with 5–6% (w/w) sulfurous acid (H_2_SO_3_) followed by oven drying at 70 °C for 24 h and at 105 °C for 1 h. Tin cups (pretreated with 1:1 hexane and acetone solution and heated to 200 °C) were used to load the sediment samples into an Elemental Analyzer (Flash EA 1112 HT, Thermo Fisher Scientific). Analysis of empty tin cups showed negligible TN and TOC. TN and TOC were estimated from five-point standard curves using flour standard containing 2.31% N and 44.39% C.

### Cell enumeration

This was done to assess the size of the microbial community and for D:L-amino acid modelling. For the sediment cores from the Iceland Basin, the Faeroe Bank, the Labrador Sea and the Godthåbsfjord, vegetative cells were quantified by fluorescence microscopy after the cells had been separated from the sediment matrix. The cell separation procedure was based on the protocol of ref. 72 and presented in ref. 73. Briefly, the cell separation procedure consists of multiple rounds of chemical detachment (detergent mix and methanol) and mechanical detachment (sonication, 3 × 10 s at 10% power). After detachment, cells were separated from the sediment matrix by density centrifugation with Nycodenz solution (50% w/v; AXIS-SHIELD PoC AS). Cell extracts were pooled and filtered onto a black polycarbonate membrane (25 mm, GTBP, 0.2 µm-pore size) and stained with DAPI-solution. A minimum of 400 cells was enumerated on at least 12 fields of view with an epifluorescence microscope (Axiovert 200 M Zeiss, Germany). To minimize contamination (introduction of external bacteria), all materials and reagents were filter-sterilized (0.2 μm) and/or autoclaved.

Cell enumeration for samples from Aarhus Bay and the Peru Margin is described in refs 6 and 33, respectively.

### Estimating the concentrations of THAA-N in microbial bio- and necromass

This was done to estimate the sizes of the different THAA-N pools in the sediment. The concentrations of sedimentary total hydrolysable amino acids (THAA) were measured directly by HPLC (see above). The concentration of THAA-N was then calculated based on the number of N atoms in each of the measured amino acids. The amount of THAA-N in the total vegetative cells, in the total endospores, and in the microbial necromass (Suppl. Fig. S3) was not measured directly but calculated as follows: subdivision of THAA-N into pools of vegetative cells and endospores was performed by multiplying microbial abundance with a cell-specific THAA-N content of 0.274 fmol cell^−1^ for both vegetative cells and endospores. This factor was derived from a cell-specific amino acid-carbon content of 1.03 fmol cell^−1^ and a C:N ratio in THAA of 3.76 (ref. 14). The concentration of THAA-N in microbial necromass was calculated from the difference between the total measured sedimentary THAA-N concentrations and the THAA-N in microbial biomass (vegetative cells + endospores). Notably, these calculations are based on the following three assumptions: (*i*) the cell-specific DPA concentration in endospores is the same independent of the site, (*ii*) the cell-specific carbon content for vegetative cells and endospores is the same independent of the site, and (*iii*) the C:N ratio in THAA of vegetative cells and endospores is the same independent of the site.

To test whether necromass amino acids were of prokaryotic origin or the remains of deposited eukaryotic detritus, we used the diagnostic ratio of the amino acids glycine and serine (Gly:Ser) (Suppl. Fig. S5a). This ratio was shown to be a source indicator of amino acids^74^. The Gly:Ser ratios were >2 throughout all sediment cores and increased slightly with sediment age. Prokaryotes were shown to have a Gly:Ser ratio of >2, while diatoms and phytoplankton have Gly:Ser ratios of ~0.7 and ~1.0, respectively (ratios are based on data from ref. 74 that were compiled by ref. 6. Based on our literature research, it is possible that the phytoplankton may have included large cyanobacteria although they are prokaryotes). This indicates that the bulk of sedimentary amino acids are not of eukaryotic, but of prokaryotic origin. In addition, the low GlcN:GalN ratios (0.7–3) throughout most of the samples (in a few of the oldest samples, the GlcN:GalN ratio was up to 9, which might be a diagenetic imprint) indicate bacterial reworking of primary amino sugars (Suppl. Fig. S5b). GlcN:GalN ratios are typically <3 in bacteria, while marine phytoplankton and zooplankton that produce chitin (e.g. copepods) have relatively high ratios (>14)^75^.

### DNA extraction and quantitative PCR

DNA extraction and q-PCR was performed to calculate the relative abundance of Bacteria (calculated as the abundance of Bacteria over the abundance of Bacteria plus Archaea), which is an input parameter to the D:L-amino acid model. The analysis was only done on the cores from the Labrador Sea (SA13-ST3-20G), the Godthåbsfjord (SA13-ST6-40G) the Iceland Basin (DA12-11/2-GC01) and the Faeroe Bank (DA12-11/2-GC03). For the sediment cores at the sites M1 and M5 in Aarhus Bay, and sites 1227, 1229 and 1230 from the Peru Margin, no qPCR data were available. For those cores, we assumed equal contributions of Bacteria and Archaea as recently suggested^11^.

The total nucleic acids were extracted from 0.5–1.0 g (wet weight) of sediment with a combination of enzymatic pretreatments and FastDNA©kit for Soil (MP Biomedicals)^76^. Following these pretreatments, the FastDNA©kit was applied according to the manufacturer’s instructions with an additional bead-beating step for 40 s at 50 Hz after the addition of sodium phosphate buffer and MT buffer. Quantitative PCR (qPCR) was performed on the DNA extracts to quantify the total number of bacterial and archaeal 16 S rDNA gene copies. The qPCR reaction mixture contained 10 μl of 2 × concentrated LightCycler® 480 SYBR Green I Master (Roche), 2 μl of BSA (10 mg ml^−1^), 1 μl of each primer, and 2 μl of DNA template for a total reaction of 20 μl. The amplification conditions were 30 s at 95 °C (5 min in the first cycle), 30 s at annealing temperature, 15 s at 72 °C for elongation for 45 cycles, and ended with the creation of a melting curve. The bacterial primers were Bac908F and 1075R^77^ (annealing temperature 60 °C), and the archaeal primers were Arch915F^78^ and Arch1059R^79^ (annealing temperature 55 °C). Reaction and thermal cycling conditions as well as qPCR standards were described previously^80^. Triplicate reactions were performed for each DNA extract. The relative abundance of Bacteria and Archaea was calculated from bacterial and archaeal 16 S rDNA gene copies after correction for cellular gene copy numbers. We assumed that Bacteria and Archaea have 4 and 2 copies of the 16S rDNA gene per cell, respectively^81^.

### D:L-amino acid racemization modelling of microbial activity

**Figure 7:**
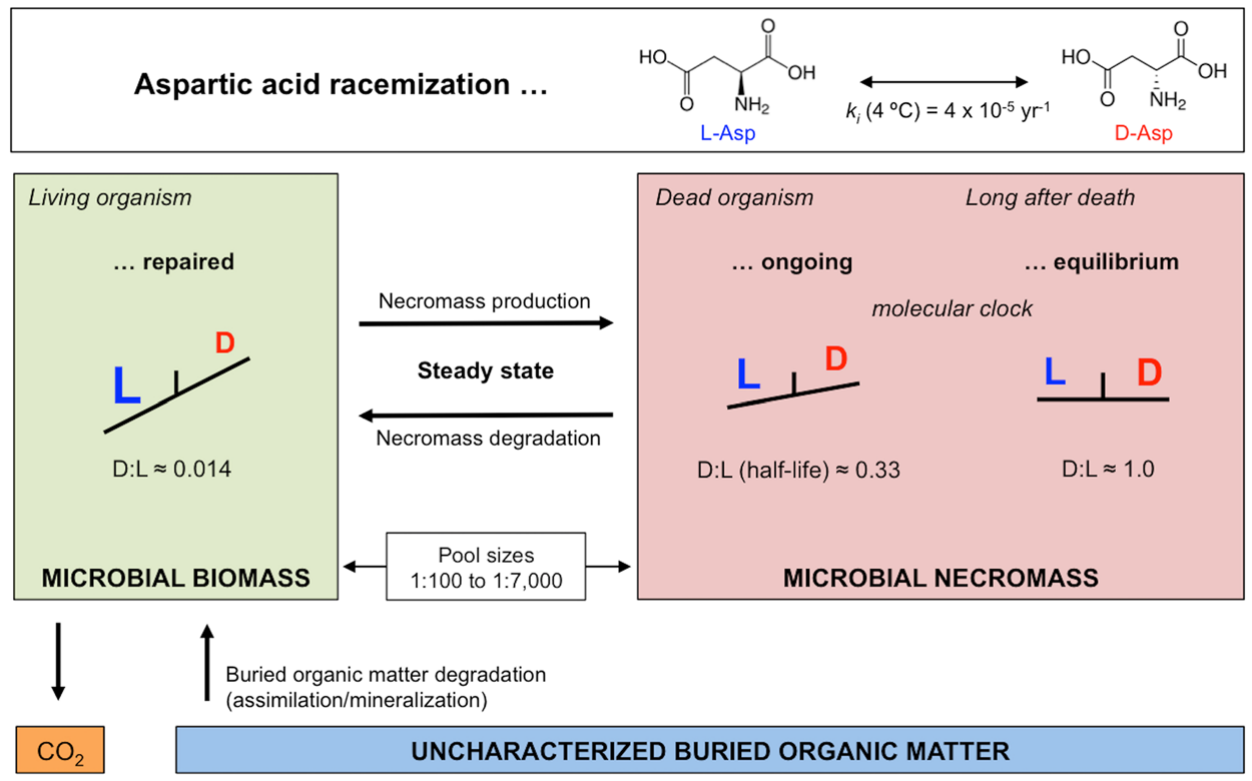
Scheme of the D:L-amino acid model. Conceptual scheme showing D:L-Asp ratios in bio- and necromass as well as different pools of amino acids with indications of the amino acid-cycling between pools. Pools are microbial biomass (vegetative cells + endospores), microbial necromass (necromass amino acids) and uncharacterized buried organic matter (not amino acids). Modified after ref. 5.

To estimate microbial biomass and necromass turnover times as well as amino acid-carbon (THAA-C) oxidation rates, we used an improved version the D:L-amino acid model developed by ref. 5. The conceptual model and a flow chart showing the parameters of the model and their connections are shown in Fig. 7 and Suppl. Fig. S6, respectively. The model is based on the built-in molecular clock of the amino acid Asp, which due to racemization alternates between the L- and D-isomeric configurations over timescales of thousands of years at low *in-situ* temperatures. Using the model, we calculated the balance between abiotic chemical racemization and biological turnover of Asp.

The model is based on the following two basic assumptions: (*i*) the microbial biomass is in quasi-steady state, and (*ii*) all Asp in microbial necromass is equally reactive.

As a consequence, the D:L-amino acid model was only applied to sediment where microbial biomass was at quasi-steady state on relevant timescales, i.e. vegetative cell abundance was relatively constant within the depth intervals used in the model. Typically, near-surface samples had to be omitted because vegetative cell abundance decreased rapidly and was therefore not in steady state. Similarly, SMTZ samples (Peru Margin) were omitted due to a steep increase in vegetative cell abundance.

To test whether all Asp in the microbial necromass is equally reactive (for example, within the necromass there could be a certain amount of Asp that is highly reactive and a certain amount of Asp that is less reactive), we compared the concentrations of Asp to concentrations of THAA in all samples (Suppl. Fig. S7). Since Asp was linearly related to the total sedimentary THAA over the full spectrum of concentrations encountered in the present study (*R* ^2^ = 0.94), we conclude that Asp is equally reactive as THAA in all samples, and hence, there is functionally only one pool of Asp in necromass. Since microbial necromass accounted for >95% of total THAA-N, it was considered appropriate to use the total Asp pool to evaluate the reactivity of Asp in necromass.

For accurate model calculations, sedimentary D:L-Asp ratios should deviate from ratios found in pure biomass, but also from ratios that would be expected from abiotic racemization only. D:L-amino acid modelling was only performed on samples that fulfilled this requirement.

Input parameters to the D:L-amino acid model that were empirically determined for each sample were the abundances of vegetative cells and endospores, the sedimentary concentrations of THAA, total Asp and D- and L-Asp, and the fraction of Bacteria over Bacteria + Archaea (this fraction was only determined for four of the nine cores; for the remaining cores, we assumed equal contributions of Bacteria and Archaea as recently suggested^11^).

Input parameters that were taken from literature values were the cell-specific amino acid-carbon content (we used the same value for vegetative cells and endospores), the cell-specific D:L-Asp ratio in subsurface microorganisms, and the racemization rate constant of Asp, k_i(Asp)_. Since k_i(Asp)_ is strongly dependent on temperature, the bottom water temperatures at the coring locations or downcore sediment temperatures were measured and translated into the corresponding k_i(Asp)_.

Because of strict quality controls in all of our HPLC analyses, we are confident that the uncertainty of the input parameters that are based on quantifications of amino acids or DPA in our lab (concentrations of THAA and Asp in sediment, the sedimentary and cellular D:L-Asp ratios, the cell-specific THAA-C content, the k_i(Asp)_, and the endospore abundance) is low. Since higher uncertainties can be expected from vegetative cell counts, the data presented here are mean values of 2–3 replicate samples.

In our recent work, we have provided new and different data on the following input parameters to the D:L-amino acid model: (*i*) the racemization rate constant, k_i(Asp)_, of sedimentary protein-bound Asp^12, 13^ instead of free Asp^28^, (*ii*) a mean cell-specific amino acid-carbon (THAA-C) content for sub-seafloor vegetative cells of 1.03 fmol THAA-C per cell^14^ instead of 4.04 fmol THAA-C per cell^5^, and (*iii*) a mean cell-specific D:L-Asp ratio in subsurface microorganisms of 0.014^15^ instead of 0.086^5^. These improved input parameters were used together with qPCR-based estimates of the fractions of Bacteria and Archaea in the samples. The fraction of Bacteria among the total microbial community may have implications for the model since Bacteria contain small amounts of D-amino acids in their cell wall complex, whereas Archaea do not contain such structural D-amino acids. Importantly, these D-amino acids are a product of bacterial anabolism and cell synthesis of cell wall complexes and are not a product of random abiotic racemization.

With this study, the mathematical model itself was improved in the following way: we included both racemization from the L- to the D-form of Asp and from the D- to the L-form. In an earlier version of the model, the back reaction of the D-form to the L-form was not taken into account^5, 6^. The factor by which the degradation rate of microbial necromass had been overestimated by not including the back reaction from D-Asp to L-Asp is up to 2 for the million-year-old samples from the Peru Margin, (see Supplementary Information for more details).

The new formula to calculate the microbial necromass degradation rate, *r*, which takes into account the back reaction from D- to L-Asp, is:

$$r=-{k}_{i(Asp)}\times \,[{{\rm{NM}}}_{{\rm{Asp}}}]\times \frac{({B}_{Asp}-1)\times ({A}_{Asp}+1)\,}{({B}_{Asp}-{A}_{Asp})}$$

A detailed model description and the full mathematical formulations of the D:L-amino acid model can be found in the Supplementary Information of ref. 5. The detailed derivation of the new formula given above can be found in the Supplementary Information.

## Electronic supplementary material

Supplementary Information

## References

[1] Inagaki F (2015). Exploring deep microbial life in coal-bearing sediment down to ~2.5 km below the ocean floor. Science.

[2] Kallmeyer J, Pockalny R, Adhikari RR, Smith DC, D’Hondt S (2012). Global distribution of microbial abundance and biomass in subseafloor sediment. Proc. Natl. Acad. Sci. USA.

[3] Parkes RJ (2014). A review of prokaryotic populations and processes in sub-seafloor sediments, including biosphere:geosphere interactions. Mar. Geol..

[4] Whitman WB, Coleman DC, Wiebe WJ (1998). Prokaryotes: the unseen majority. Proc. Natl. Acad. Sci. USA.

[5] Lomstein BA, Langerhuus AT, D’Hondt S, Jørgensen BB, Spivack A (2012). Endospore abundance, microbial growth and necromass turnover in deep subseafloor sediment. Nature.

[6] Langerhuus AT (2012). Endospore abundance and D:L-amino acid modeling of bacterial turnover in holocene marine sediment (Aarhus Bay). Geochim. Cosmochim. Acta.

[7] Røy H (2012). Aerobic microbial respiration in 86-million-year-old deep-sea red clay. Science.

[8] Hoehler TM, Jørgensen BB (2013). Microbial life under extreme energy limitation. Nat. Rev. Microbiol..

[9] Cowie GL, Hedges JI (1992). Sources and reactivities of amino acids in a coastal marine environment. Limnol. Oceanogr..

[10] Wakeham, S. G. & Lee, C. Production, transport, and alteration of particulate organic matter in the marine water column. In *Organic Geochemistry: Principles and Applications* (eds Engel, M. H. & Macko, S. A.) (Plenum Press, 1993).

[11] Lloyd KG, May MK, Kevorkian RT, Steen AD (2013). Meta-analysis of quantification methods shows that Archaea and Bacteria have similar abundances in the Subseafloor. Appl. Environ. Microbiol..

[12] Steen AD, Jørgensen BB, Lomstein B (2013). Aa. Abiotic racemization kinetics of amino acids in marine sediments. PLoS ONE.

[13] The *PLOS ONE* Staff. Correction: Abiotic racemization kinetics of amino acids in marine sediments. *PLoS ONE* **10**(4): e0123837; doi:10.1371/journal.pone.0123837 (2015). 10.1371/journal.pone.0123837 PMC4390380 25853861

[14] Braun S (2016). Size and carbon content of sub-seafloor microbial cells at Landsort Deep, Baltic Sea. Front. Microbiol..

[15] Braun S (2016). Cellular content of biomolecules in sub-seafloor microbial communities. Geochim. Cosmochim. Acta.

[16] Isaksen MF, Bak F, Jørgensen BB (1994). Thermophilic sulfate-reducing bacteria in cold marine sediment. FEMS Microbiol. Ecol..

[17] Rothfuss F, Bender M, Conrad R (1997). Survival and activity of bacteria in a deep, aged lake sediment (Lake Constance). Microb. Ecol..

[18] Fichtel J, Koster J, Rullkotter J, Sass H (2007). Spore dipicolinic acid contents used for estimating the number of endospores in sediments. FEMS Microbiol. Ecol..

[19] Lomstein BA, Jørgensen BB (2012). Pre-column liquid chromatographic determination of dipicolinic acid from bacterial endospores. Limnol. Oceanogr. Methods.

[20] de Rezende JR (2013). Dispersal of thermophilic *Desulfotomaculum* endospores into Baltic Sea sediments over thousands of years. ISME J..

[21] Henrichs SM (1992). Early diagenesis of organic matter in marine sediments: progress and perplexity. Mar. Chem..

[22] Hedges JI, Keil RG (1995). Sedimentary organic matter preservation: an assessment and speculative synthesis. Mar. Chem..

[23] Burdige DJ (2007). Preservation of organic matter in marine sediments: controls, mechanisms, and an imbalance in sediment organic carbon budgets?. Chem. Rev..

[24] Lomstein BA, Jørgensen BB, Schubert CJ, Niggemann J (2006). Amino acid biogeo- and stereochemistry in coastal Chilean sediments. Geochim. Cosmochim. Acta.

[25] Lomstein BA, Niggemann J, Jørgensen BB, Langerhuus AT (2009). Accumulation of prokaryotic remains during organic matter diagenesis in surface sediments off Peru. Limnol. Oceanogr..

[26] Lee C, Wakeham SG (1988). Organic matter in seawater. Chem. Oceanogr.

[27] Wakeham SG, Lee C, Hedges J, Hernes PJ, Peterson ML (1997). Molecular indicators of diagenetic status in marine organic matter. Geochim. Cosmochim. Acta.

[28] Bada JL (1982). Racemization of amino acids in nature. Interdiscipl. Sci. Rev..

[29] D’Hondt, S., Wang, G. & Spivack, A. J. The underground economy (energetic constraints of subseafloor life), in *Earth and Life Processes Discovered from Subseafloor Environments: A Decade of Science Achieved by the Integrated Ocean Drilling Program (IODP)* (eds Stein, R., Blackman, D. K., Inagaki, F., Larsen, H.-H.) 127–144 (Elsevier, 2014).

[30] Hernández SB, Cava F (2016). Environmental roles of microbial amino acid racemases. Environ. Microbiol..

[31] Williams KM, Smith GG (1977). A critical evaluation of the application of amino acid racemization to geochronology and geothermometry. Origins of Life.

[32] Onstott TC (2014). Does aspartic acid racemization constrain the depth limit of the subsurface biosphere?. Geobiology.

[33] Shipboard Scientific Party. Leg 201 summary. *Proc. ODP Init. Rep*. **201**, 1–81 (2003).

[34] Jørgensen BB (2011). Deep subseafloor microbial cells on physiological standby. Proc. Natl. Acad. Sci. USA.

[35] Wehmiller J, Hare PE (1971). Racemization of amino acids in marine sediments. Science.

[36] Kaiser K, Benner R (2005). Hydrolysis-induced racemization of amino acids. Limnol. Oceanogr. Methods.

[37] Kriausakul N, Mitterer RM (1978). Isoleucine epimerization in peptides and proteins: Kinetic factors and application to fossil proteins. Science.

[38] Henrichs SM, Farrington JW, Lee C (1984). Peru upwelling region sediments near 15°S. 2. Dissolved free and total hydrolyzable amino acids. Limnol. Oceanogr..

[39] Pedersen A-GU, Thomsen TR, Lomstein B, Aa., Jørgensen NOG (2001). Bacterial influence on amino acid enantiomerization in a coastal marine sediment. Limnol. Oceangr..

[40] Mitterer, R. M. Data report: D/L ratios and concentrations of selected amino acids in interstitial waters, equatorial Pacific and Peru margin, ODP Leg 201 In *Proceedings of the Ocean Drilling Program, Scientific Results*, 201 (eds Jørgensen, B. B., D’Hondt, S. L. & Miller, D. J.) 1–7 (Ocean Drilling Program, Texas A&M University, College Station TX 77845–9547, USA, 2006).

[41] Engel MH, Zumberge JE, Nagy B (1977). Kinetics of amino acid racemization in *Sequoiadendron giganteum* heartwood. Anal. Biochem..

[42] Goodfriend GA (1997). Aspartic acid racemization and amino acid composition of the organic endoskeleton of the deep-water colonial anemone *Gerardia*: Determination of longevity from kinetic experiments. Geochim. Cosmochim. Acta.

[43] Brinton KLF, Tsapin AI, Gilichinski D, McDonald GD (2002). Aspartic acid racemization and age-depth relationships for organic carbon in Siberian permafrost. Astrobiol..

[44] Ingraham, J. L., Maaløe, O. & Neidhardt, F. C. Growth of the Bacterial Cell. (Sunderland, Massachusetts: Sinauer Associates, Inc, 1983).

[45] Simon M, Azam F (1989). Protein content and protein synthesis rates of planktonic marine bacteria. Mar. Ecol. Prog. Ser..

[46] Schippers A (2005). Prokaryotic cells of the deep sub-seafloor biosphere identified as living bacteria. Nature.

[47] Biddle JF (2006). Heterotrophic Archaea dominate sedimentary subsurface ecosystems off Peru. Proc. Natl. Acad. Sci. USA.

[48] D’Hondt S (2004). Distributions of microbial activities in deep subseafloor sediments. Science.

[49] Wellsbury P, Mather I, Parkes RJ (2002). Geomicrobiology of deep, low organic carbon sediments in the Woodlark Basin, Pacific Ocean. FEMS Microbiol. Ecol..

[50] Engelhardt T, Orsi WD, Jørgensen BB (2015). Viral activities and life cycles in deep subseafloor sediments. Environ. Microbiol. Rep..

[51] Schink, B. In Biology of Anaerobic Microorganisms (ed. Zehnder, A. J. B.) 771–846 (Wiley-Interscience, 1988).

[52] Schink, B. & Stams, A. J. M. In *The Prokaryotes: An Evolving Electronic Resource for the Microbiological Community* (eds Dworkin, M. *et al*.) 309–335 (Springer, 2002).

[53] van den Vossenberg JLCM, Ubbink-Kok T, Elferink MGL, Driessen AJM, Konings WN (1995). Ion permability of the cytoplasmic membrane limits the maximum growth temperature of bacteria and archaea. Mol. Microbiol..

[54] Jørgensen BB, Parkes RJ (2010). Role of sulphate reduction and methane production by organic carbon degradation in eutrophic fjord sediments (Limfjorden, Denmark). Limnol. Oceanogr..

[55] Detmers J, Brüchert V, Habicht KS, Kuever J (2001). Diversity of sulfur isotope fractionations by sulfate-reducing prokaryotes. Appl. Environ. Microbiol..

[56] Knoblauch C, Jørgensen BB (1999). Effect of temperature on sulfate reduction, growth rate, and growth yield in five psychrophilic sulfate-reducing bacteria from Arctic sediments. Environ. Microbiol..

[57] Knoblauch C, Jørgensen BB, Harder J (1999). Community size and metabolic rates of psychrophilic sulfate-reducing bacteria in Arctic marine sediments. Appl. Environ. Microbiol..

[58] Tarpgaard IH, Boetius A, Finster K (2006). Desulfobacter psychrotolerans sp. nov., a new psychrotolerant sulfate-reducing bacterium and descriptions of its physiological response to temperature changes. A. van Leeuw. J. Microb..

[59] Starnawski P (2017). Microbial community assembly and evolution in subseafloor sediment. Proc. Natl. Acad. Sci. USA.

[60] LaRowe DE, Amend JP (2015). Power limits for microbial life. Front. Microbiol..

[61] Zinser ER, Kolter R (1999). Mutations enhancing amino acid catabolism confer a growth advantage in stationary phase. J. Bacteriol..

[62] Finkel SE (2006). Long-term stationary survival during stationary phase: evolution and the GASP phenotype. Nat. Rev. Microbiol..

[63] Shipboard Scientific Party. Benthic Research at Sea, Dana 12-11, Reykjavik – Hirtshals. *Cruise Report*, 1–38 (2012).

[64] Shipboard Scientific Party. Godthåbsfjord system and the West Greenland shelf with ‘R/V Sanna’, 11.-16. August 2013. *Cruise Report*, 1–10 (2013).

[65] Malmberg SA (1962). Schichtung und Zirculation in den Südländischen Gewässern. Kieler Meeresforschungen.

[66] Reimer PJ (2013). IntCal13 and Marine13 Radiocarbon Age Calibration Curves 0–50,000 Years cal BP. Radiocarbon.

[67] Ramsey CB (2008). Deposition models for chronological records. Quat. Sci. Rev..

[68] Ramsey CB (2009). Bayesian analysis of radiocarbon dates. Radiocarbon.

[69] Lindroth P, Mopper K (1979). High-performance liquid-chromatographic determination of subpicomole amounts of amino-acids by precolumn fluorescence derivatization with ortho-phthaldialdehyde. Anal. Chem..

[70] Mopper K, Furton KG (1991). Extraction and analysis of polysaccharides, chiral amino-acids, and SFE-extractable lipids from marine POM. Mar. Part.: Anal. Charact..

[71] Guldberg LB, Finster K, Jørgensen NOG, Middelboe M, Lomstein B (2002). Aa. Utilization of marine sedimentary dissolved organic nitrogen by native anaerobic bacteria. Limnol. Oceanogr..

[72] Kallmeyer J, Smith DC, Spivack AJ, D’Hondt S (2008). New cell extraction procedure applied to deep subsurface sediments. Limnol. Oceanogr. Methods.

[73] Glombitza C (2015). Formate, acetate and propionate as substrates for sulfate reduction in sub-arctic sediments of Southwest Greenland. Front. Microbiol..

[74] Keil, R. G., Tsamakis, E. & Hedges, J. I. Early diagenesis of particulate amino acids in marine systems In *Perspectives in Amino Acid and Protein Geochemistry* (eds Goodfriend, G. A., Collins, M. J., Fogel, M. L., Macko, S. A. & Wehmiller, J. F.) 69–82 (Oxford University Press, 2000).

[75] Benner R, Kaiser K (2003). Abundance of amino sugars and peptidoglycan in marine particulate and dissolved organic matter. Limnol. Oceanogr..

[76] Kjeldsen KU (2007). Diversity of sulfate-reducing bacteria from an extreme hypersaline sediment, Great Salt Lake (Utah). FEMS Microbiol. Ecol..

[77] Ohkuma M, Kudo T (1998). Phylogenetic analysis of the symbiotic intestinal microflora of the termite Cryptotermes domesticus. FEMS Microbiol. Lett..

[78] Cadillo-Quiroz H (2006). Vertical profiles of methanogenesis and methanogens in two contrasting acidic peatlands in central New York State, USA. Environ. Microbiol..

[79] Yu Y, Lee C, Kim J, Hwang S (2005). Group-specific primer and probe sets to detect methanogenic communities using quantitative real-time polymerase chain reaction. Biotechnol. Bioeng..

[80] Nielsen MB, Kjeldsen KU, Lever MA, Ingvorsen K (2014). Survival of prokaryotes in a polluted waste dump during remediation by alkaline hydrolysis. Ecotoxicol..

[81] Suna D-L, Jianga X, Wub QL, Zhoua N-Y (2013). Intragenomic Heterogeneity of 16S rRNA Genes Causes Overestimation of Prokaryotic Diversity. Appl. Environ. Microbiol..

