# Supplementary material for: Microbial turnover times in the deep seabed studied by amino acid racemization modelling
Source: Sci Rep. 2017 Jul 18;7:5680. doi: 10.1038/s41598-017-05972-z (PMC5516024; doi:10.1038/s41598-017-05972-z)
Supplement: Supplementary file 1 — Supplementary Information [file 41598_2017_5972_MOESM1_ESM.doc]

**Supplementary Information**

**Microbial turnover times in the deep seabed studied by amino acid racemization modelling**

Stefan Braun^a,+^, Snehit S. Mhatre^a,+^, Marion Jaussi^a^, Hans Røy^a^, Kasper U. Kjeldsen^a^, Christof Pearce^c,d^, Marit-Solveig Seidenkrantz^c,e^, Bo B. Jørgensen^a^, and Bente Aa. Lomstein^a,b*^

^a^*Center for Geomicrobiology, Department of Bioscience, Aarhus University, Ny Munkegade 114, 8000 Aarhus C, Denmark*

^b^*Section for Microbiology, Department of Bioscience, Aarhus University, Ny Munkegade 114, 8000 Aarhus C, Denmark*

^c^*Center for Past Climate Studies, Department of Geoscience, Aarhus University, 8000 Aarhus C, Denmark*

^d^*Department of Geological Sciences and Bolin Centre for Climate Research, Stockholm University, 10691 Stockholm, Sweden*

^e^*Arctic Research Center, Department of Bioscience, Aarhus University, 8000 Aarhus C, Denmark*

^*^Correspondence: Bente Aa. Lomstein, Center for Geomicrobiology and Section for Microbiology, Department of Biosciences, Aarhus University, Ny Munkegade 114, 8000 Aarhus-C, Denmark. bente.lomstein@bios.au.dk

^+^These authors share first authorship

**1. D:L-amino acid racemization modelling of bacterial activity**

To estimate biomass and necromass turnover times and THAA-C oxidation rates, we used the D:L-amino acid model developed by ref. 1. A complete list of D:L-amino acid model nomenclature is given in Supplementary Table S2. The conceptual model and a flow chart showing the parameters of the model and their connections are shown in Fig. 7 and Suppl. Fig. S6, respectively.

The model is based on the built-in molecular clock of aspartic acid (Asp), which due to racemization alternates between the D- and L-isomeric configurations over timescales of thousands of years at low *in-situ* temperatures (1-16 °C). Using the model, we calculated the balance between abiotic chemical racemization and biological turnover of Asp.

The model is based on the following two basic assumptions and can only be applied to sediment samples where

- the microbial biomass is in quasi-steady state
- all Asp in microbial necromass is equally reactive

(Tests on whether these pre-requisites were fulfilled for our samples were done as described in the Methods Summary.)

Input parameters to the D:L-amino acid model that were empirically determined for each sample (see also Suppl. Fig. S6) were

- the abundances of vegetative cells and endospores
- the sedimentary concentrations of THAA, total Asp, and D- and L-Asp
- the fraction of Bacteria over Bacteria+Archaea (this fraction was only determined for four of the nine cores; for the remaining cores, we assumed equal contributions of Bacteria and Archaea as recently suggested^2^).

Input parameters literature values were taken for (see also Suppl. Fig. S6) were

- the cell-specific amino acid-carbon (THAA-C) content (we used the same value for vegetative cells and endospores, which was 1.03 fmol THAA-C per cell with a C:N ratio in THAA of 3.76)^3^
- the cell-specific D:L-Asp ratio in subsurface microorganisms (D:L-Asp = 0.014)^4^
- the racemization rate constant of Asp, k_i(Asp)_. Since k_i(Asp)_ is strongly dependent on temperature, the bottom water temperatures at the coring locations or downcore sediment temperatures were measured and translated into the corresponding k_i(Asp)_.

With this study, the model itself was improved in the following way: we included both racemization from the L- to the D-form of Asp and from the D- to the L-form. In an earlier version of the model, it had been accounted for the conversion from L to D only^1,5^.

The detailed model description and mathematical formulations can be found in the Supplementary Information of ref. 1. In the following, we present the mathematical changes made to the model by including both racemization from the L- to the D-form of Asp and from the D- to the L-form (all changes compared to ref. 1 are underlined in the text).

We consider the necromass pool. Loss rates denote transfer out of the necromass pool (e.g. into the biomass pool). Production rates denote transfer into the necromass pool (e.g. death of biomass, i.e. production of necromass).

The loss rate of L-Asp into D-Asp due to racemization, RL_loss, is


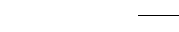
 (1)

where k_i(Asp)_ is the racemization rate constant of Asp at the *in-situ* temperature, [NM_Asp_] is the total concentration of Asp in microbial necromass, and B_Asp_ is the steady state D:L-Asp ratio in microbial necromass, which is estimated from the measured D:L-Asp ratio of the sediment sample, the mean D:L-Asp ratio in the microbial community, A_Asp_, and the fraction of THAA-C associated with living cells, f_Live_ (this equation is equal to equation (26) in ref. 1):


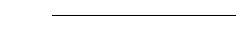
 (2)

The mean D:L-Asp ratio in the living microbial community, A_Asp_, is calculated as follows (this equation is equal to equation (27) in ref. 1):


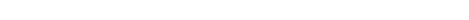
 (3)

where f_archaea_ is the archaeal contribution to living THAA-C, and f_bacteria+endospores_ is the contribution of THAA-C from bacterial cells and endospores to the total living THAA-C pool. D:L-Asp_archaea_ is zero because archaea do not contain D-amino acids in their cell walls complex. D:L-Asp_bacteria_ is the D:L-Asp ratio in pure bacterial biomass.

The production rate of L-Asp due to racemization, RL_prod, is equal to the concentration of D-Asp in necromass, [D-Asp_NM_], times the racemization rate constant for Asp, k_i(Asp)_:


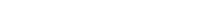
 (4)

The concentration of D-Asp in microbial necromass is the total concentration of Asp in necromass minus the fraction that is L-Asp (this equation is equal to equation (1) in ref. 1):


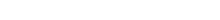
 (5)

Microbial necromass has a calculated steady-state D:L-Asp ratio = B_Asp_.

We set
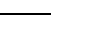
 (6)


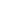


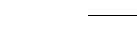
 (7)


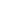
 by substituting equation (5) by equation (7)


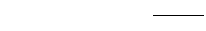
 (8)


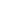


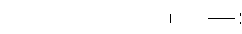
 (9)


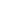


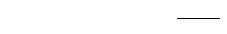
 (10)


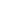


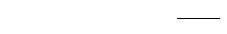
 (11)

By substituting equation (11) into equation (4), the production rate of L-Asp due to racemization is therefore:


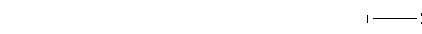
 (12)

The net loss rate of L-Asp due to racemization, RL_net loss, is thus the sum of production and loss:


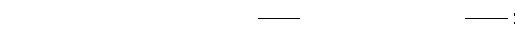
 (13)


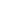


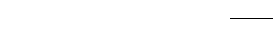
 (14)

Living microbial biomass has a mean D:L-Asp ratio = A_Asp_. The necromass production rate (not due to racemization, but due to death of biomass) for L-Asp, r_Lnp_, is the total necromass Asp production rate, r_np_, times the fraction of L-Asp in Living Mass (living biomass, LM, is equal to vegetative cells plus endospores) (this equation is equal to equation (8) in ref. 1):


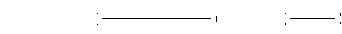
 (15)

Where
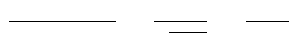
 (16)

The degradation rate for L-Asp in necromass (not due to racemization, but due to microbial activity), r_Lnd_, is the total Asp degradation rate, r_nd_, times the fraction of L-Asp in necromass (this equation is equal to equation (17) in ref. 1):


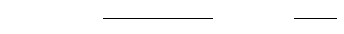
 (17)

We assume quasi-steady state. For L-Asp in necromass this means that production minus degradation plus the net loss of L-Asp due to racemization is equal to zero:


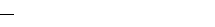
 (18)


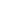


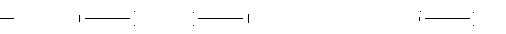
 (19)

We now rearrange and set r_np_ = r_nd_ = r.


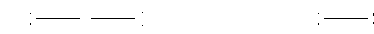
 (20)

where,


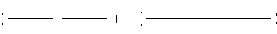
 (21)

Substitution of equation (21) into equation (20) gives:


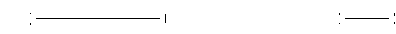
 (22)


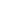


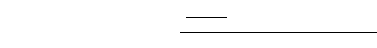
 (23)

When all pools are in steady state, the microbial necromass degradation rate (r_nd_ = r) can be estimated from the following equation, which is the new equation accounting for both the racemization from the L- to the D- form and from the D- to the L-form of Asp:


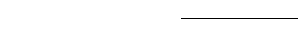
 (24)

where k_i(Asp)_ is the chemical racemization rate constant at the corresponding temperature, [NM_Asp_] is the concentration of Asp in the necromass, A_Asp_ is the mean D:L-Asp ratio in the microbial biomass, and B_Asp_ is the D:L-Asp ratio in microbial necromass. The difference to the earlier version of the model^1^ is the factor (B_Asp_-1). When the turnover of necromass is fast and most of the Asp pool is in the L-form, then B_Asp_ is small and the factor (B_Asp_-1) is close to -1 (which corresponds to the equation (24) in ref. 1 used to calculate the steady state necromass turnover rate, r). When the turnover is very slow, however, and B_Asp_ grows to a significant value between 0 and 1, then the factor (B_Asp_-1) becomes numerically smaller than 1 and the correctly calculated turnover rate becomes correspondingly smaller.

For example, in the samples from the Peru Margin, the D:L-Asp ratios in necromass up to a sediment age of 5 million years are ~0.2-0.45. Thus, the factor (B_Asp_-1) is -0.8 to -0.55. Therefore, the actual degradation rate of microbial necromass is only 55-80% of what had been estimated according to the original formula in ref. 1.

The necromass turnover time, T_NM_, can be calculated from the necromass degradation rate (this equation is equal to equation (25) in ref. 1):


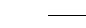
 (25)

The turnover times of vegetative cells, T_b_, can be estimated from the total carbon in vegetative cells (assuming cell THAA-C = 55% total cell carbon^6^), and the THAA-C degradation rate, r_THAA-C_:


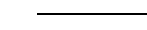
 (26)

where THAA-C in cells is the cell-specific amino acid-carbon content (we used a mean THAA-C content of 1.03 fmol THAA-C cell^-1^ specific for sub-seafloor cells)^3^, and r_THAA-C_ is the concentration of THAA-C in necromass divided by the necromass turnover time:


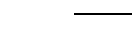
 (27)

**2. Supplementary Figures**

**Supplementary Fig. S1 | Map showing the sediment core locations.** The map was generated using the free and open source geographic information system software QGIS version 2.4.0 ([https://qgis.org](https://qgis.org/)). Land polygons are downloaded from the Natural Earth website (<http://www.naturalearthdata.com/downloads/110m-physical-vectors/110m-land/>) and are in the Public Domain (<http://www.naturalearthdata.com/about/terms-of-use/>).


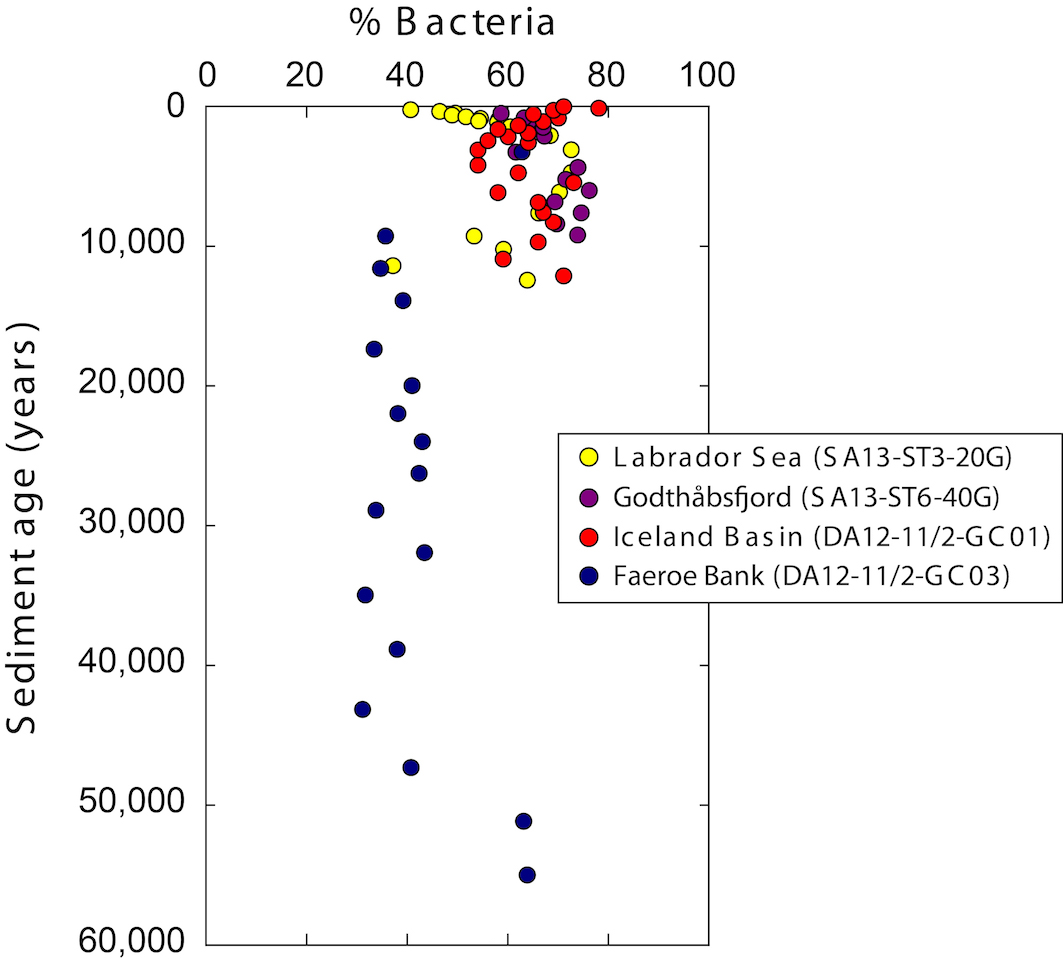


**Supplementary Fig. S2 | Relative abundance of Bacteria (calculated as the abundance of Bacteria over Bacteria+Archaea) estimated from qPCR analysis.** For the sediment cores at the sites M1 and M5 in Aarhus Bay, and sites 1227, 1229 and 1230 from the Peru Margin, no qPCR data were available. For those cores, we assumed equal contributions of Bacteria and Archaea as recently suggested^2^


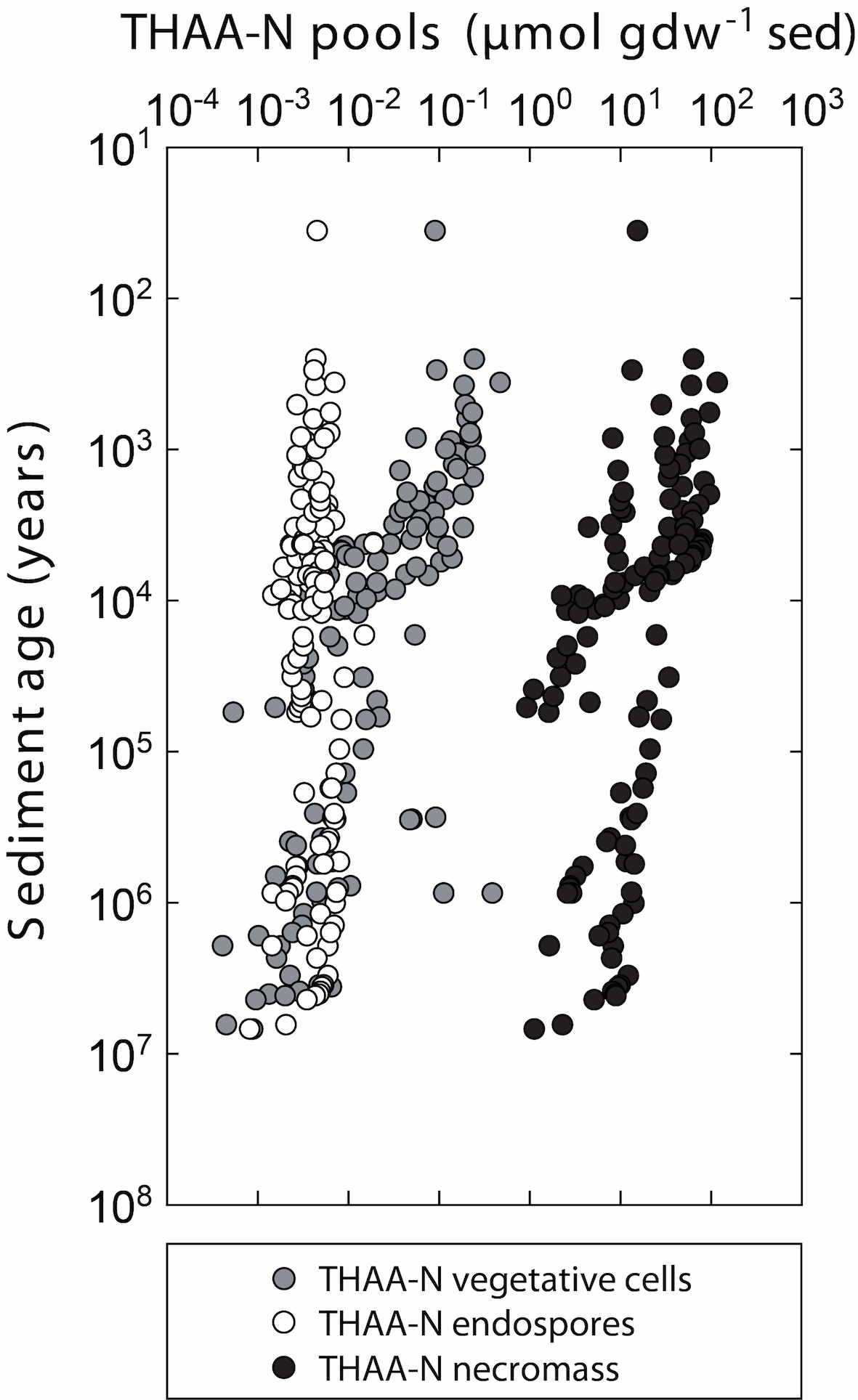


**Supplementary Fig. S3 | Subdivision of sedimentary THAA-N into pools of vegetative cells, endospores, and microbial necromass.** Total sedimentary THAA-N consists of >95% microbial necromass. The unit ‘per gram dry weight sediment’ is abbreviated with ‘gdw^-1^ sed’. Note that the concentrations of THAA-N in vegetative cells, endospores and microbial necromass (the sum of which is the sedimentary THAA-N) were not measured directly, but estimated based on measurements on vegetative cell and endospore abundances and literature conversion factors for the cellular content of THAA-N (see Methods Summary for details on how the calculations were performed).


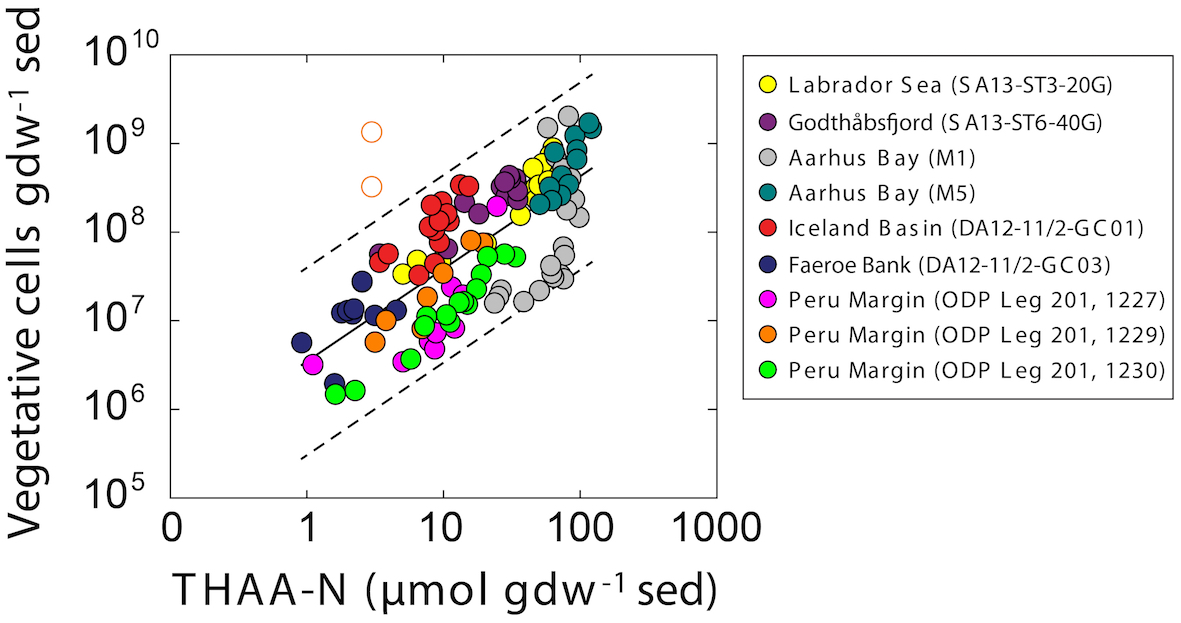


**Supplementary Fig. S4 | Relationship between vegetative cell abundance and sedimentary concentrations of THAA-N.** Regression lines show total microbial vegetative cells (solid line; log_10_[cells] = 6.537 × log_10_[THAA-N] + 1.045, *N* = 119, *R*^2^ = 0.54, *P* < 0.0001, least squares analysis) and 95% prediction interval (dashed lines). Open circles denote data points that have been removed from regression analysis (e.g. sulphate-methane transition zones). The unit ‘per gram dry weight sediment’ is abbreviated with ‘gdw^-1^ sed’.


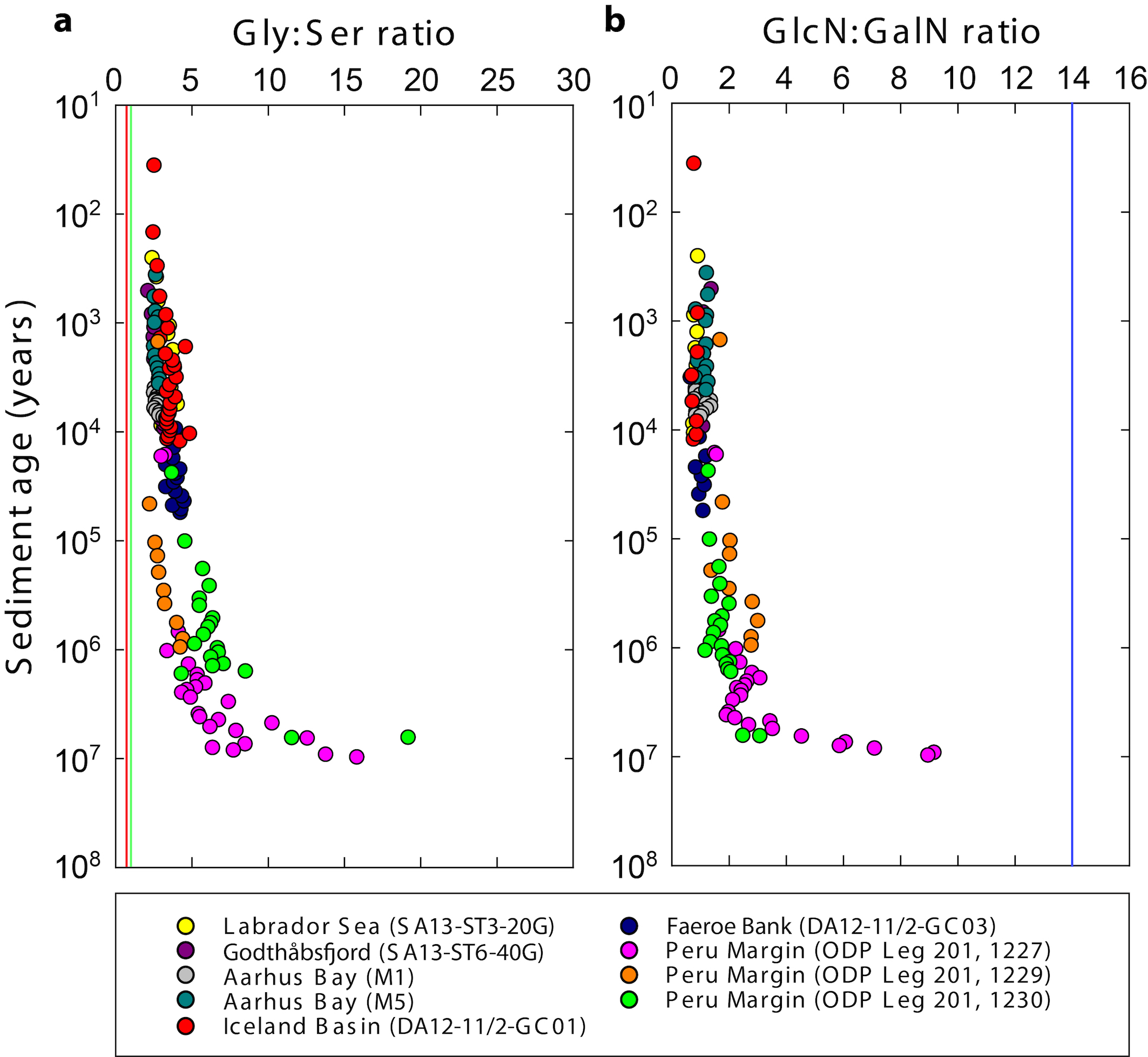


**Supplementary Fig. S5 | Diagnostic ratios of (a) the amino acids glycine and serine (Gly:Ser) and (b) the amino sugars glucosamine and galactosamine (GlcN:GalN) to determine the origin of the labile organic compounds.** Prokaryotes typically have Gly:Ser and GlcN:GalN ratios of >2 (ratios are based on data from ref. 7 that were compiled by ref. 5) and <3 (ref. 8), respectively. Red and green lines in (**a**) denote Gly:Ser ratios found in diatoms (0.7) and phytoplankton (1.0), respectively (ratios are based on data from ref. 7 that were compiled by ref. 5). Blue line in (**b**) denotes GlcN:GalN ratio of ~14 found in zooplankton (chitin-rich materials, e.g. copepods)^8^.


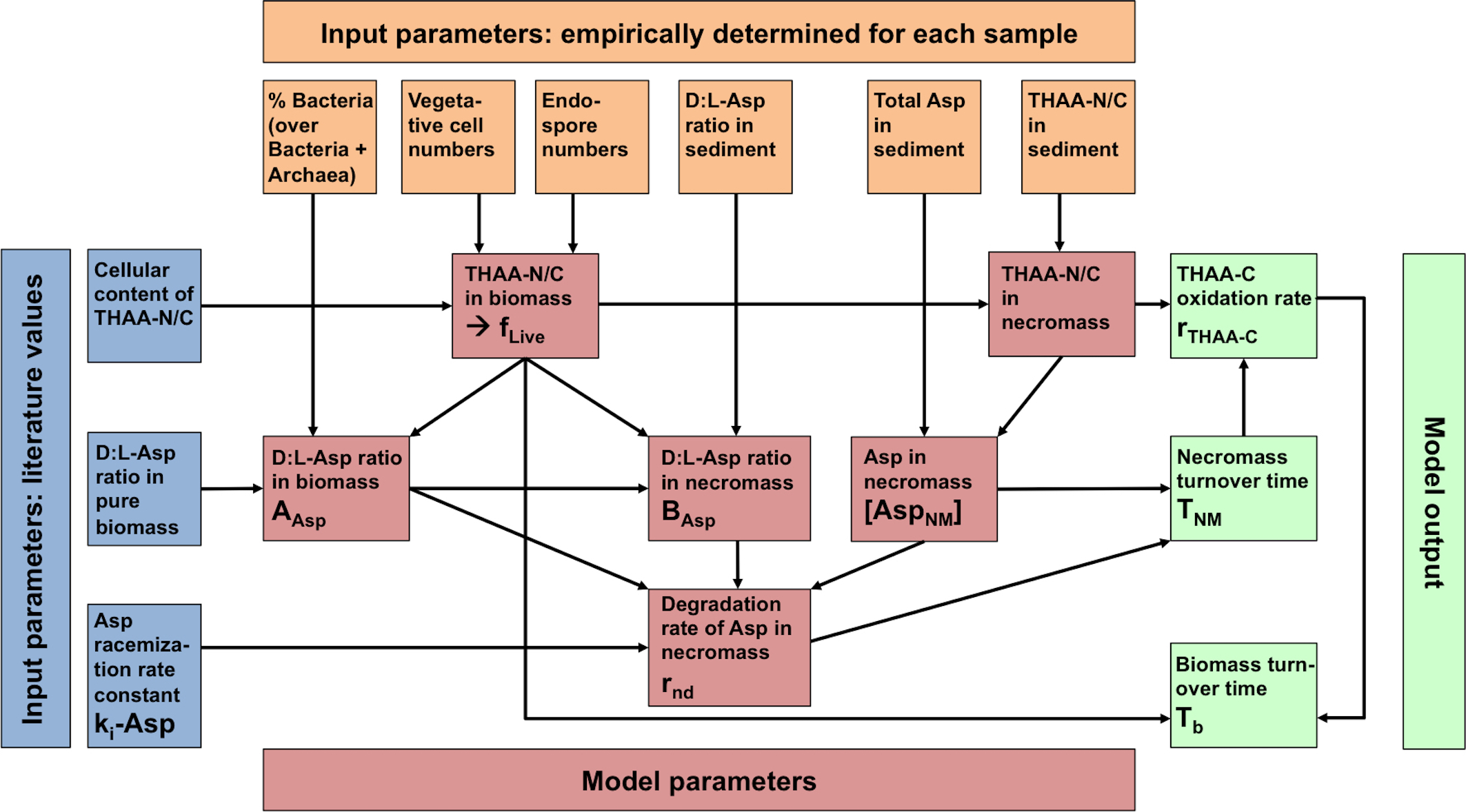


**Supplementary Fig. S6 | Flow chart of the D:L-amino acid model showing the different parameters of the model and their connections.** Arrows denote calculation steps.


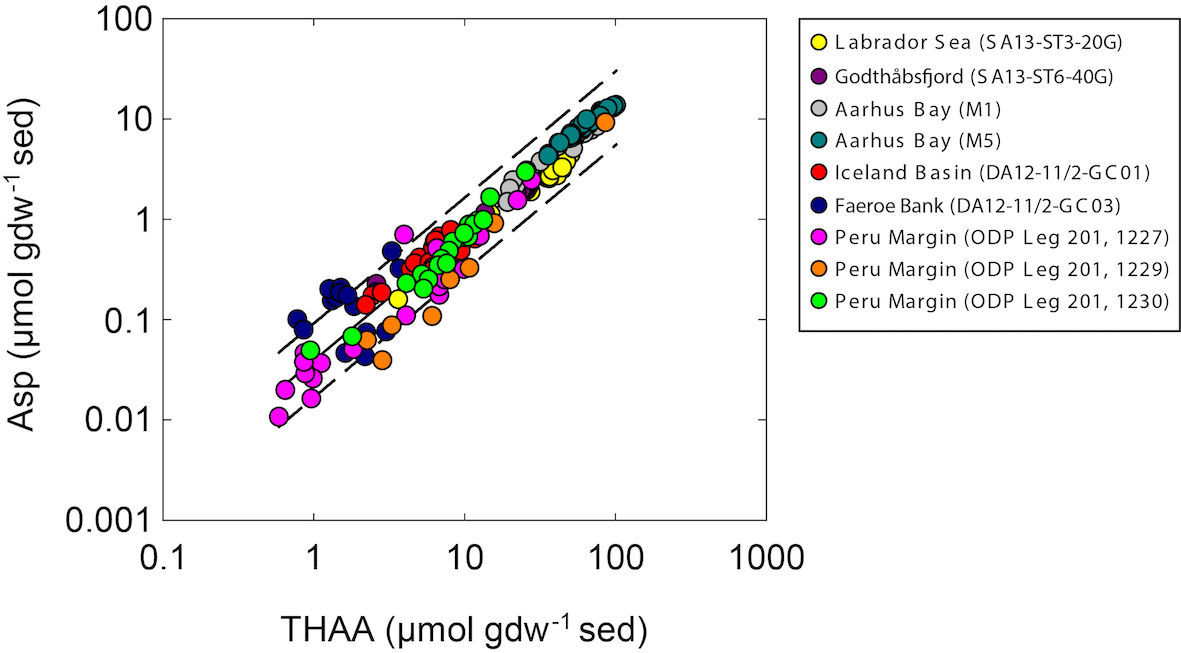


**Supplementary Fig. S7 | Relationship between Asp and THAA.** Regression lines show [Asp] versus [THAA] (solid line; log_10_[Asp] = 1.592 × log_10_[THAA] + 1.256, *N* = 180, *R*^2^ = 0.94, *P* < 0.0001, least squares analysis) and 95% prediction interval (dashed lines). The unit ‘per gram dry weight sediment’ is abbreviated with ‘gdw^-1^ sed’.

**3. Supplementary Tables**

**Supplementary Table S1 | Overview of the sediment core locations, coordinates, water depths, core lengths, and bottom water temperatures.**

| **Site/Core-ID** | **Location** | **Latitude** | **Longitude** | **Water depth (m)** | **Core length (m)** | **Bottom water temperature (°C)** |
| --- | --- | --- | --- | --- | --- | --- |
| SA13-ST3-20G | Labrador Sea | 64°26.743’N | 52°47.649’W | 498 | 5.9 | 4.4 |
| SA13-ST6-40G | Godthåbsfjord | 64°29.060’N | 50°42.324’W | 389 | 5.6 | 1.8 |
| DA12-11/2-GC01 | Iceland Basin | 61°36.536’N | 20°42.164’W | 2,120 | 4.2 | 3 (ref. 9) |
| DA12-11/2-GC03 | Faeroe Bank | 60°46.943’N | 9°47.624’W | 742 | 5.9 | 7.6 |
| M1 | Aarhus Bay | 56°07.066’N | 10°20.793’E | 15 | 10.9 | 7.8 (ref. 5) |
| M5 | Aarhus Bay | 56°06.20’N | 10°27.47’E | 28 | 6.1 | 7.8 (ref. 5) |
| ODP Leg 201, Site 1227 | Peru Margin | 8°59.49’S | 79°54.35’W | 427 | 151.1 | 8.6 (ref. 10) |
| ODP Leg 201, Site 1229 | Peru Margin | 10°58.60’S | 77°57.46’W | 151 | 194.4 | 13.4 (ref. 10) |
| ODP Leg 201, Site 1230 | Peru Margin | 9°06.78’S | 80°35.01’W | 5,086 | 278.3 | 1.7 (ref. 10) |

**Supplementary Table S2 |** D:L-amino acid model nomenclature.

| **Property** | **Explanation** | **Quantification** | **Numerical value in this study** |
| --- | --- | --- | --- |
| ***Observational model input*** |  |  |  |
| Vegetative cell numbers | Abundance of vegetative cells in the sediment | Epifluorescence microscopy | ~10^6^-10^9^ cells gdw^-1^ sediment |
| Endospore numbers | Abundance of endospores in the sediment | Sedimentary DPA concentrations | ~10^6^-10^8^ endospores gdw^-1^ sediment |
| % Bacteria | Fraction of Bacteria over Bacteria+Archaea | qPCR | 30-80% |
| D:L-Asp ratio in sediment | D:L-Asp ratio in sediment | HPLC | <0.5 |
| Total Asp in sediment | Concentration of Asp in the sediment | HPLC | ~0.01-10 µmol gdw^-1^ sediment |
| THAA-N in sediment | Concentration of amino acid-nitrogen in the sediment | HPLC | ~0.5-120 µmol gdw^-1^ sediment |
| THAA-C in cells | Amino acid-carbon content of cells | Literature value^3^ | 1.03 fg THAA-C cell^-1^ |
| D:L-Asp_bacteria_ | D:L-Asp ratio in pure bacterial biomass | Literature value^4^ | 0.014 |
| k_i(Asp)_ | Racemization rate constant of Asp at site-specific temperature | Literature value^11,12^ | ~3 × 10^-5^ – 3 × 10^-4^ |
|  |  |  |  |
| ***Model output*** |  |  |  |
| T_NM_ | Turnover time of microbial necromass | Model calculation | ~1000-23,000 years |
| r_THAA-C_ | Degradation rate of amino acid-carbon in microbial necromass | Model calculation | ~0.6-180 nmol gdw^-1^ sediment yr^-1^ |
| T_b_ | Turnover time of microbial biomass | Model calculation | ~0.5-120 years |
|  |  |  |  |
| ***Other model parameters*** |  |  |  |
| NM | Microbial necromass | Model calculation | No primary model output |
| Asp_NM_ | Asp in microbial necromass | Model calculation | No primary model output |
| L-Asp_NM_ | L-Asp in microbial necromass | Model calculation | No primary model output |
| D-Asp_NM_ | D-Asp in microbial necromass | Model calculation | No primary model output |
| L-Asp_LM_ | L-Asp in Living Mass (cells+endospores) | Model calculation | No primary model output |
| D-Asp_LM_ | D-Asp in Living Mass (cells+endospores) | Model calculation | No primary model output |
| RL_loss | The microbial necromass loss rate of L-Asp into D-Asp due to racemization | Model calculation | No primary model output |

*(Table continued)*

| RL_prod | The microbial necromass production rate of L-Asp due to racemization of D-Asp into L-Asp | Model calculation | No primary model output |
| --- | --- | --- | --- |
| RL_net loss | The net loss of L-Asp in microbial necromass due to racemization is the sum of production and loss | Model calculation | No primary model output |
| r_Lnp_ | Production rate of L-Asp in microbial necromass | Model calculation | No primary model output |
| r_Dnp_ | Production rate of D-Asp in microbial necromass | Model calculation | No primary model output |
| r_np_ | Production rate of Asp in microbial necromass | Model calculation | No primary model output |
| r_Lnd_ | Degradation rate of L-Asp in microbial necromass | Model calculation | No primary model output |
| r_Dnd_ | Degradation rate of D-Asp in microbial necromass | Model calculation | No primary model output |
| r_nd_ | Degradation rate of Asp in microbial necromass | Model calculation | No primary model output |
| f_Live_ | Fraction of amino acid-carbon in living microbial biomass from bacteria and endospores | Model calculation | No primary model output |
| A_Asp_ | D:L-Asp ratio in living microbial biomass | Model calculation | No primary model output |
| B_Asp_ | D:L-Asp ratio in microbial necromass | Model calculation | No primary model output |
| f_archaea_ | The archaeal contribution to living amino acid-carbon | Model calculation | No primary model output |
| f_bacteria+endospores_ | The contribution to living amino acid-carbon from bacteria and endospores | Model calculation | No primary model output |

**References**

1. Lomstein, B. Aa., Langerhuus, A. T., D’Hondt, S., Jørgensen, B. B. & Spivack, A. Endospore abundance, microbial growth and necromass turnover in deep subseafloor sediment. *Nature* **484,** 101-104 (2012).
2. Lloyd, K. G., May, M. K., Kevorkian, R. T. & Steen, A. D. Meta-analysis of quantification methods shows that Archaea and Bacteria have similar abundances in the Subseafloor. *Appl. Environ. Microbiol.* **79,** 7790-7799 (2013).
3. Braun, S. *et al*. Size and carbon content of sub-seafloor microbial cells at Landsort Deep, Baltic Sea. *Front. Microbiol.* **7,** 1375; 10.3389/fmicb.2016.01375 (2016).
4. Braun, S. et al. Cellular content of biomolecules in sub-seafloor microbial communities. *Geochim. Cosmochim. Acta* **188,** 330-351 (2016).
5. Langerhuus, A. T. *et al*. Endospore abundance and D:L-amino acid modeling of bacterial turnover in holocene marine sediment (Aarhus Bay). *Geochim. Cosmochim. Acta* **99,** 87-99 (2012).
6. Ingraham, J. L., Maaløe, O. & Neidhardt, F. C. *Growth of the Bacterial Cell*. (Sunderland, Massachusetts: Sinauer Associates, Inc, 1983).
7. Keil, R. G., Tsamakis, E. & Hedges, J. I. Early diagenesis of particulate amino acids in marine systems in Perspectives in Amino Acid and Protein Geochemistry (eds. Goodfriend, G. A., Collins, M. J., Fogel, M. L., Macko, S. A. & Wehmiller, J. F.) 69-82 (Oxford University Press, 2000).
8. Benner, R, & Kaiser, K. Abundance of amino sugars and peptidoglycan in marine particulate and dissolved organic matter. *Limnol. Oceanogr*. **48,** 118-128 (2003).
9. Malmberg, S. A. Schichtung und Zirculation in den Südländischen Gewässern. *Kieler Meeresforschungen* **18,** 3-28 (1962).
10. Shipboard Scientific Party. Leg 201 summary. *Proc. ODP Init. Rep.* **201,** 1-81 (2003).
11. Steen, A. D., Jørgensen, B. B. & Lomstein, B. Aa. Abiotic racemization kinetics of amino acids in marine sediments. *PLoS ONE* **8**(8): e71648; 10.1371/journal.pone.0071648 (2013).
12. The PLOS ONE Staff. Correction: Abiotic racemization kinetics of amino acids in marine sediments. *PLoS ONE* **10**(4): e0123837; 10.1371/journal.pone.0123837 (2015).
